# An Enhanced Phenology Dataset for Global Drylands from 2001 to 2019

**DOI:** 10.1038/s41597-025-05519-2

**Published:** 2025-07-09

**Authors:** Yuqi Dong, Yu Zhou, Li Zhang, Feng Tian, Qiaoyun Xie, Yiyang Chen, Linlin Ruan, Bo Zhang

**Affiliations:** 1 International Research Center of Big Data for Sustainable Development Goals, Beijing, 100094 People’s Republic of China; 2 Key Laboratory of Digital Earth Science, Aerospace Information Research Institute, Chinese Academy of Sciences, Beijing, 100094 People’s Republic of China; 3 University of Chinese Academy of Sciences, Beijing, 100094 People’s Republic of China; 4 Department of Environmental Systems Science, ETH Zurich, Zurich, 8092 Switzerland; 5 School of Integrative Plant Science, Cornell University, Ithaca, 14850 NY United States of America; 6 Hubei Key Laboratory of Quantitative Remote Sensing of Land and Atmosphere, School of Remote Sensing and Information Engineering, Wuhan University, Wuhan, China; 7 School of Engineering, The University of Western Australia, Perth, WA 6009 Australia; 8 Centre for Water and Spatial Science, The University of Western Australia, Perth, WA 6009 Australia

**Keywords:** Ecosystem ecology, Plant ecology, Biogeography, Carbon cycle, Climate-change ecology

## Abstract

Drylands dominate the interannual variability of global carbon sink, and phenology is a key driver of carbon sequestration. However, accurately retrieving dryland phenology from satellite data remains challenging due to sparse and heterogeneous vegetation. Existing land surface phenology (LSP) products exhibit low accuracy in drylands due to coarse spatiotemporal data sources and algorithms optimized for other ecosystems. Here we present the Global Dryland Phenology Dataset (GDPD) for 2001–2019, derived from daily 500-m two-band Enhanced Vegetation Index using MODIS NBAR data and an improved retrieval algorithm with dynamic, pixel-wise amplitude thresholds. GDPD covers 88.4% of global drylands, compensating for missing regions in other LSP products. GDPD shows strong agreements with *in-situ* phenology from PhenoCam GCC (SOS: *r* = 0.88; EOS: *r* = 0.72) and physiology from flux tower GPP (SOS: *r* = 0.96; EOS: *r* = 0.90). We highlight the importance of high-resolution data in improving dryland phenology retrieval. This dataset improves our understanding of how dryland ecosystems respond to climate change and supports the development of Earth system models.

## Background & Summary

Accurately characterizing vegetation phenology in drylands is essential since drylands dominate the interannual variability of the global carbon sink^1,2^ and their phenology plays an important role in determining carbon sequestration and understanding terrestrial ecosystem structure and function^3^. Vegetation phenology characterizes the timing of seasonal biological events such as germination, flowering, and abscission^4^, reflecting the response of terrestrial ecosystems to global climate and hydrological changes and has been recognized as a key biological indicator of climate change impacts^5,6^.

Despite significant advancements in remote sensing techniques^7,8^, accurately retrieving dryland vegetation phenology from satellite images remains challenging due to the sparse coverage and high heterogeneity of dryland vegetation. Current global land surface phenology (LSP) products performe poorly in drylands compared to other ecosystems^9,10^. For example, the six major LSP products showed greate variation in onset dates in the continental United States with sparse vegetation cover^11^. Additionally, LSP products often fail to detect phenological events in drylands, such as the western United States^10^ and Australia^12^, or exhibit weak correlations with *in-situ* observations^13^.

One reason for the poor performance is that some global LSP products applied fixed amplitude thresholds across diverse biome types without considering biome-specific variations (Table 1), resulting in neglect of subtle changes in dryland ecosystems. For example, a seasonal cycle is considered valid by the MODIS Land Cover Dynamics Product (MCD12Q2) when the seasonal amplitude of the two-band enhanced vegetation index (EVI2) is greater than or equal to 0.1 and greater than or equal to 35% of the maximum EVI2 variation exhibited over the three-year period^14^. As a result, MCD12Q2 missed a large number of pixels with low seasonal amplitude in dryland ecosystems^15–17^.

**Table 1:** Information of existing global LSP products.

Product name	MODIS Phenology^86^	VIIRS/NPP Land Surface Phenology^87^	AVHRR-based Phenology^88^	Vegetation Index and Phenology^38^
Abbreviation	MCD12Q2	VNP22Q2	AVH12	VIPPHEN
Data source	MODIS NBAR-EVI2	VIIRS NBAR-EVI2	AVHRR EVI2	AVHRR and MODIS EVI2
Temporal coverage	2001–2021	2013–2022	1982–2019	1981–2014
Resolutions of data source	500 m, 16-day	500 m, daily	0.05 degrees, daily	0.05 degrees, daily
Interpolation and smoothing filter methods	Penalized cubic spline interpolation and the Savitzky-Golay filtering method	Moving average of the two neighboring high-quality values, hybrid piecewise logistic model	Spline interpolation and the Savitzky-Golay filtering method	Temporal smoothing algorithm
Retrieval methods for phenological metrics	Amplitude threshold (15%)	Extreme values of curvature change values	Amplitude threshold (20%); first-, second-, and third-order derivatives; relative change rate; curvature change rate	Amplitude threshold (35%)
Main limitations in dryland phenology	1. The estimated greenup and dormancy are not accurate for some high-latitude dryland regions. 2. Pixel retrieval fails in drylands due to the low amplitude of EVI2 variation and fixed threshold.	1. Short temporal span. 2. Growth curves of dryland ecosystems generally do not follow the S-shaped curve generated by the logistic function^89^. 3. The data cycles are defined on four key phenological metrics without concern for the order of their appearance, so there will be regions where the greenup onset is later than the dormancy onset.	1. Data source with coarse spatial resolution. 2. SOS is later than EOS in some regions. 3. No retrieval of multiple growing seasons.	1. Data source with coarse spatial resolution. 2. Phenology is not considered below when EVI2 < 0.08 and a minimum seasonal EVI2 = 0.03 is required.

Another reason is that spatiotemporal scale effects pose challenges to accurate retrieval of phenology^18^, especially in highly heterogeneous dryland ecosystems^19^, with mixed biomes including grasses, shrubs, and trees, as well as the high coverage of bare soil. Each component exhibits a distinct response to climatic drivers, especially precipitation, which is difficult to detect and distinguish from current LSP products using coarse spatial resolution data that are generally greater than 0.05 degrees^20,21^. Furthermore, it has been shown that the effect of spatial resolution on phenological indicators is complex and depends on the characteristics of the local biomes^22^. For example, Liu *et al*.^23^ found that phenological metrics retrieved from data at different spatial scales were similar in homogeneous grassland sites but less comparable at heterogeneous savanna sites. Coarse temporal resolutions, e.g., 8-day and 16-day adopted by existing LSP products, are unable to accurately capture the peak of vegetation growth in drylands, where vegetation responds rapidly to concentrated rainfall over short periods and exhibits high interannual variability compared to other ecosystems^24^. As temporal resolution decreases, fewer effective observations are available to characterize the growing season in dryland ecosystems, resulting in increased uncertainty in phenological metrics estimates^25^, particularly during the greening phase when EVI2 changes more rapidly than during the senescence phase^26^.

To our knowledge, several studies have focused on improving the accuracy of vegetation phenology retrieval in drylands^27–30^. Notably, Xie *et al*.^12^ modified the fixed seasonal amplitude threshold used in MCD12Q2 by applying pixel-wise EVI2 averages, resulting in an improved success rate of phenological metric retrieval in arid and semi-arid ecosystems in Australia. In this study, we further enhanced and extended this algorithm to generate the global dryland phenology dataset (GDPD) from 2001 to 2019 by replacing the original fixed amplitude threshold with a pixel-wise dynamic threshold. We compared GDPD against other commonly used LSP products in Table 1 and validated the results using PhenoCam GCC and flux tower GPP data. We further performed varying spatiotemporal resolution experiments at two sites with different vegetation types to assess the effects of spatial and temporal resolutions on dryland phenology retrieval. Our dataset outperformed existing datasets regarding both date accuracy and spatial coverage, and we highlighted both its values and limitations.

## Methods

### Study area

**Fig. 1:**
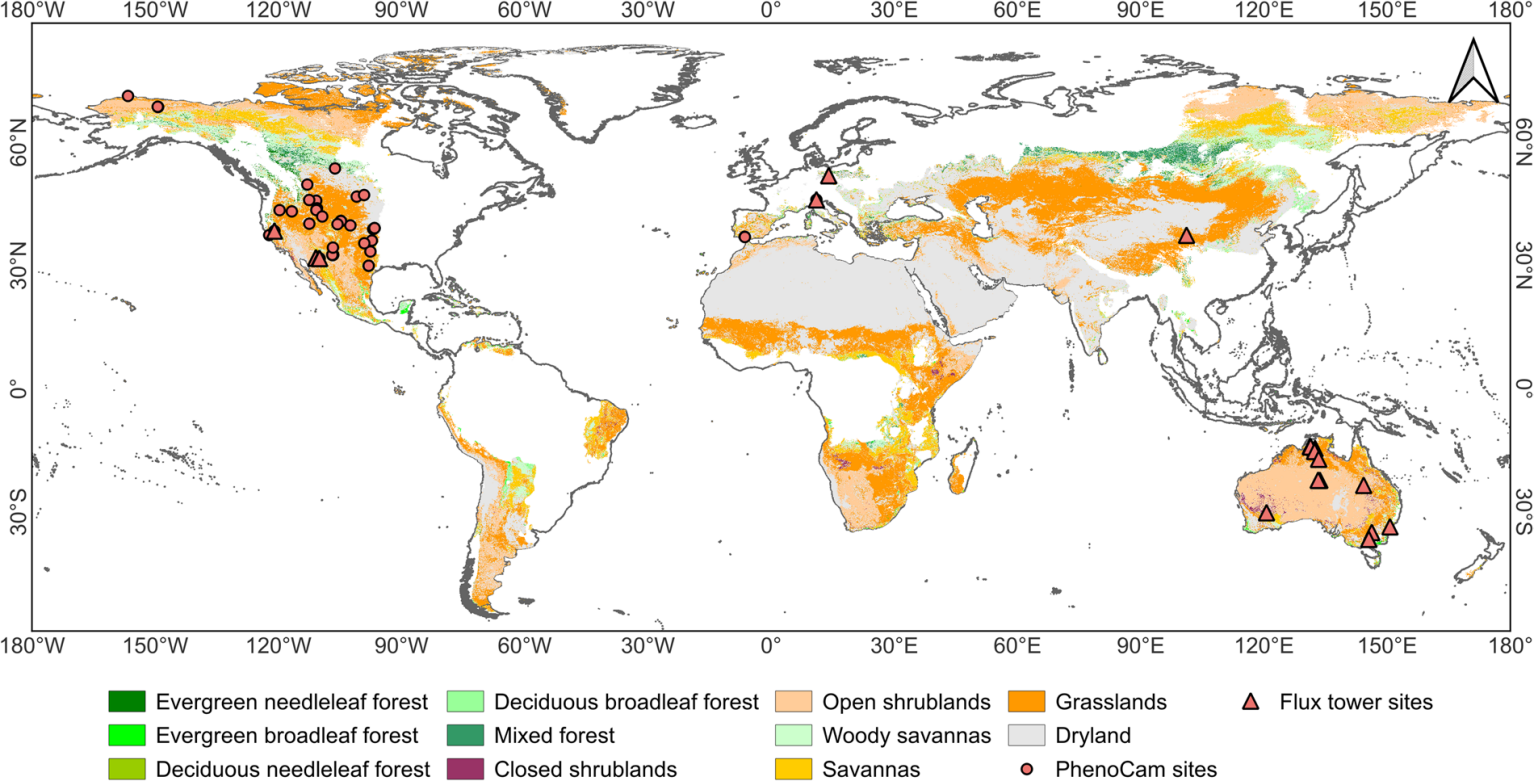
Study area and the geographic distribution of the PhenoCam and flux tower sites used in this study.

Drylands are defined as areas where the Aridity Index (AI, the ratio of mean annual precipitation to mean annual potential evapotranspiration) is less than 0.65^31^. In this study, we extracted dryland regions based on the Global Aridity Index and Potential Evapotranspiration Database version 3 at 30-arcsecond spatial resolution (https://csidotinfo.wordpress.com/2019/01/24/global-aridity-index-and-potential-evapotranspiration-climate-database-v3)^32^. Vegetation types were identified using the MODIS Land Cover Type (MCD12Q1 v061) product (10.5067/MODIS/MCD12Q1.061)^33^, which follows the International Geosphere-Biosphere Programme (IGBP) classification system at 500-m spatial resolution. To minimize anthropogenic influences on vegetation phenology, we excluded cropland, urban areas, and built-ups in this study. Study areas with consistent vegetation cover were identified by analyzing land cover maps from 2001, 2009, and 2018, and selecting pixels where vegetation types remained unchanged across these years (Fig. 1), thereby minimizing the impact of land cover change on the comparability of phenological metrics across periods.

### Two-band enhanced vegetation index (EVI2)

The MODIS MCD43A4 (v061) product provides daily Nadir Bidirectional Reflectance Distribution Function-Adjusted Reflectance (NBAR) at 500-m resolution using composites of Terra and Aqua MODIS data (10.5067/MODIS/MCD43A4.061)^34^, which reduces the impact of solar zenith angles on phenology retrieval^35^. We performed quality control on each band using the quality assurance (QA) flags, retaining only high-quality pixels (QA = 0). The daily two-band enhanced vegetation index (EVI2, Eq. 1) was calculated for 2001–2019 to retrieve phenological metrics. EVI2 minimizes the influence of soil background and atmospheric interference^36^, and provides reliable monitoring of vegetation dynamics with low uncertainty^37^. To reduce noise, we excluded EVI2 values less than or equal to 0.08, which were considered to be non-vegetated or inactive areas (e.g., cloud shadows, snow, or bare soil)^38^. Univariate spline interpolation was used to fill in missing data in the EVI2 time series, followed by the Savitzky–Golay (SG) filtering method to smooth the data^39^.

1 $${EVI}2=2.5\ast \frac{{NIR}-{Red}}{{NIR}+2.4\ast {Red}+1}$$

where *NIR* is near-infrared band reflectance and *Red* is red band reflectance.

### Estimation of vegetation phenological metrics

**Fig. 2:**
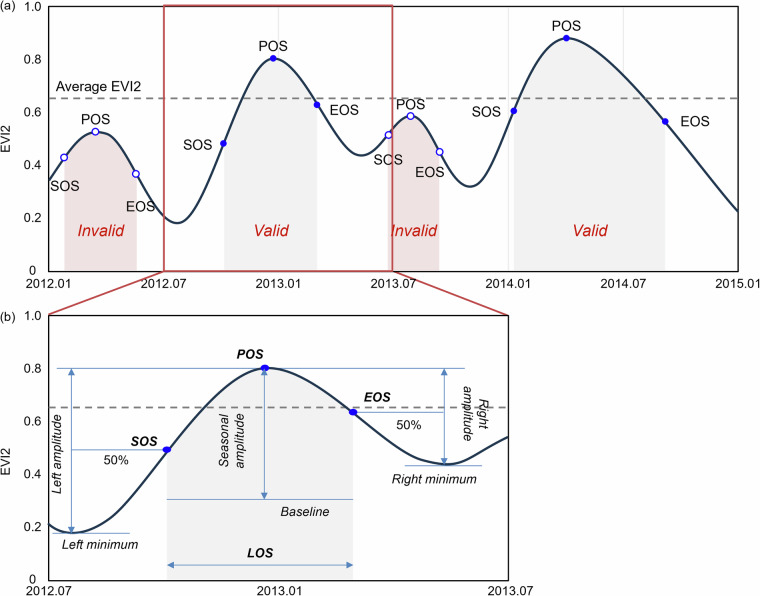
Conceptual diagram to illustrate the (**a**) improved algorithm for retrieving (**b**) phenological metrics from EVI2 time series.

The conceptual diagram of the phenological metrics retrieval algorithm is shown in Fig. 2. Based on the EVI2 time series, we retrieved phenological metrics accounting for up to two growing seasons per year. We defined the start of the growing season (SOS) as the point when the EVI2 value increased to 50% of the left-side amplitude (Fig. 2), and the end of the growing season (EOS) as the point when the EVI2 decreased to 50% of the right-side amplitude for each growing season. 50% amplitude reduces the susceptibility of EVI2 values to background soil signals during early or late stages of vegetation growth^40^. The length of the growing season (LOS) was the difference between EOS and SOS. The peak of the growing season (POS) corresponded to the time when EVI2 reached the maximum value during the growing season. The baseline was the average of the left and right minimum values, and the seasonal amplitude was the difference between the maximum EVI2 value and the baseline.

To avoid spurious peaks, we modified the fixed amplitude threshold used by the MCD12Q2 and instead used dynamic pixel-wise EVI2 averages, i.e., we retrieved the phenological metrics only when the peak EVI2 value was greater than or equal to the average EVI2 of the retrieval window (i.e., the target year with six months before and after) for each pixel. This improvement enhanced detection of more growing seasons with varying amplitudes across time and biomes, which has been shown to improve the retrieval ratio of phenological metrics in Australian arid and semi-arid ecosystems^12,41^.

To assess the impact of the spatio-temporal resolution selection of data sources on phenology retrieval, we conducted a varying spatiotemporal resolution experiment at two dryland sites with distinct vegetation types, including ES-LJu dominated by open shrublands (OSH) (10.18140/FLX/1440157)^42^, and IT-Ro1 dominated by deciduous broadleaf forest (DBF) (10.18140/FLX/1440174)^43^. We resampled the original EVI2 (500 m, daily; referred to as EVI2_O_) into coarse temporal resolution (500 m, 16-day; EVI2_CT_) and coarse spatial resolution (0.05 degrees, daily; EVI2_CS_). Additionally, we compared the daily GPP time series for each site to evaluate the ability of different spatial and temporal resolutions of EVI2 data captured photosynthetic dynamics.

To explore the robustness of the algorithm on future sensor series, we replaced MCD43A4 with the daily 500-m VIIRS NBAR product as the input data source, and tested the improved amplitude threshold phenology retrieval algorithm. Detailed methodology and results are included in Text S6.

### Accuracy assessment

To evaluate the performance of GDPD and the other four LSP products, we calculated their retrieval ratio (*RR*, Eq. 2) separately for each year from 2001 to 2019. A larger *RR* value indicates a higher success ratio of phenological metrics retrieval.

2 $${RR}=\frac{m}{M}\times 100 \% $$

where *m* is the number of pixels with retrieved phenological metrics, and *M* is the number of total pixels in the study area.

For validation, we compared the phenological metrics derived from our study (i.e., GDPD) with ground observations (PhenoCam, FLUXNET 2015 and OzFlux). Additionally, the four LSP products (MCD12Q2, VNP22Q2, AVH12, and VIPPHEN) were similarly evaluated against PhenoCam, flux data following quality control (Text S1). PhenoCam (10.3334/ORNLDAAC/1674) is a global network for tracking vegetation phenology using leverage near-surface remote sensing, primarily focused on North American terrestrial ecosystems^44^. PhenoCam cameras record three-layer JPEG images, from which we extracted mean intensity data for red, green, and blue (RGB) channels within a user-defined region of interest (ROI). To ensure high-quality data, we selected high-quality type I cameras and excluded sites lacking continuous observation for one year. Evergreen forest sites were excluded from this study, since PhenoCam does not effectively capture seasonal variation in these ecosystems^45^. In total, 169.5 site-years across 41 sites were used in this study (Fig. 1, Table S5). The green chromatic coordinate (GCC, Eq. 3) was calculated for each image within each ROI, which has been frequently used to derive near-surface time series for phenological metrics analysis^46^.

3 $${GCC}=\frac{{Green}}{{Red}+{Green}+{Blue}}$$

where *Red*, *Green*, and *Blue* respectively represent the average red, green and blue digital numbers (a measure of intensity) in each ROI. A daily time series of GCC was generated by calculating the 90th percentile of GCC values for each day based on the 30-minute observations^47^. After applying the SG filter as described earlier, we identified the SOS and EOS of the growing season by extracting 20% of the seasonal amplitude from the GCC time series^48,49^.

The flux tower GPP data (FLUXNET 2015 (https://fluxnet.org/data/fluxnet2015-dataset/) and OzFlux (https://ozflux.org.au/monitoringsites/index.html)) offers the opportunity to analyze the consistency of phenological metrics with photosynthetic activity across numerous global sites^11^, which also compensates for the sparse spatial distribution of PhenoCam sites. As with PhenoCam, we excluded evergreen forests and selected 24 sites with more than three years of high-quality carbon flux data (≥75% data completeness per year) (Fig. 1, Table S6). We used GPP estimates based on the nighttime partitioning method^50^. After smoothing the GPP time series with SG filters, we applied a 20% threshold of seasonal amplitude to define SOS and EOS for GPP. In contrast to the 50% threshold applied to EVI2 time series, the 20% threshold better captures subtle seasonal dynamics present in continuous GPP and GCC measurements, and has the highest correlation with EVI2-retrieved phenological metrics (Text S2, Fig. S1). Since only a few pixels presented two growing seasons in dryland vegetation, we only evaluated phenological metrics for the first growing season in the Results section, and those with a confirmed second season based on validation data (PhenoCam GCC or flux-tower GPP) were presented in the Supplement (Text S4).

The Taylor diagram provides a concise statistical summary of the relationship between LSP products and validation data (PhenoCam GCC and flux tower GPP data) in terms of their correlation (*r*, Eq. 4), centered root-mean-square error (*cRMSE*, Eq. 5), and the standard deviation (*SD*, Eq. 6)^51^. It visually represents the distance between an LSP product and the validation data, with shorter distances indicating higher consistency.

4 $$r=\frac{\mathop{\sum }\limits_{i=1}^{n}\left({F}_{i}-\bar{F}\right)\left({O}_{i}-\bar{O}\right)}{n{{SD}}_{O}{{SD}}_{F}}$$

5 $${cRMSE}=\sqrt{\frac{{\mathop{\sum }\limits_{i=1}^{n}(\left({F}_{i}-\bar{F}\right)\left({O}_{i}-\bar{O}\right))}^{2}}{n}}$$

6 $${{SD}}_{O}=\sqrt{\frac{{\mathop{\sum }\limits_{i=1}^{n}\left({O}_{i}-\bar{O}\right)}^{2}}{n}},{{SD}}_{F}=\sqrt{\frac{{\mathop{\sum }\limits_{i=1}^{n}\left({F}_{i}-\bar{F}\right)}^{2}}{n}}$$

where *n* is the sample size; $\bar{F}$ and $\bar{O}$ are the mean values of *F* (phenology estimates of LSP product) and *O* (site observations), respectively.

To investigate if phenology retrieval is robust across different seasonal amplitudes of EVI2, we calculated the phenological metrics errors (relative to *in-situ* observations) and regressed against seasonal amplitude. We also stratified the sites into low-, medium-, and high-amplitude groups and tested whether SOS/EOS errors differed significantly among these groups (details in Text S5).

## Data Records

GDPD provides annual 500-m vegetation phenological metrics in global drylands from 2001 to 2019 with up to two growth cycles per year, including the start of the growing season (SOS) and end of season (EOS). The unit of SOS and EOS is the day of year (DOY). The entire dataset is deposited at the open-access repository Figshare (10.6084/m9.figshare.27160602.v2^52^) in the GEOTIFF format. The file names are structured according to the file naming scheme “*GDPD*_<*year*>_<*name of phenological metrics*>_<*number of growing seasons*>.tif”. Here, *GDPD* is the abbreviation for Global Dryland Phenology Dataset, *year* is the retrieval year for phenological metrics, *name of phenological metrics* includes the start of the growing season (SOS) and the end of the growing season (EOS), and *number of growing seasons* includes the first (season1) and second (season2) growing seasons.

## Technical Validation

### Comparison of retrieval ratio of phenological metrics across global dryland

**Fig. 3:**
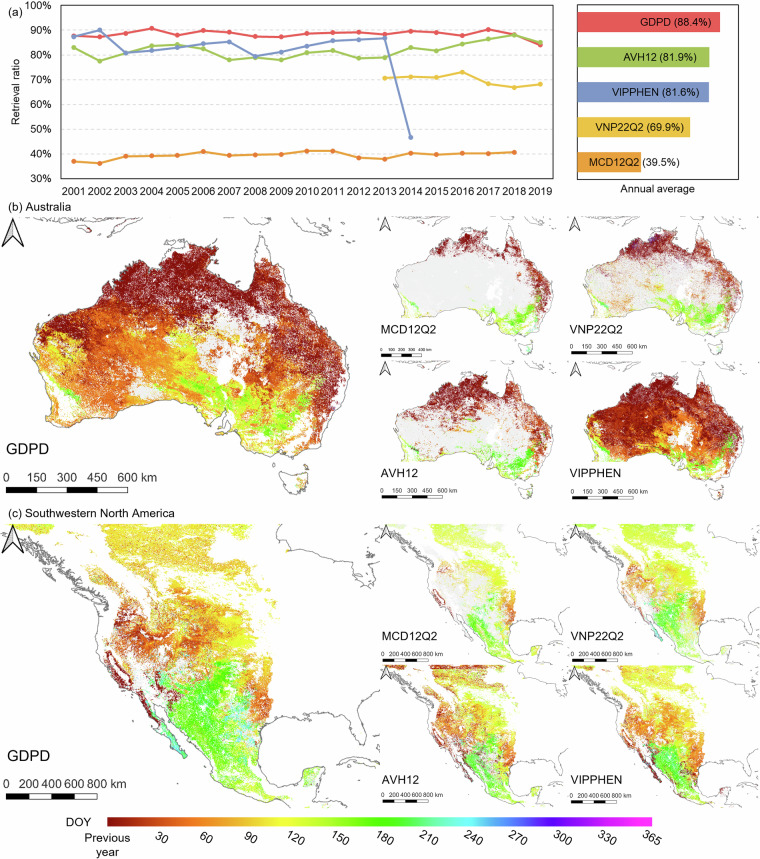
(**a**) Retrieval ratio (RR) and averages (barplot) of GDPD and the other four LSP products for global drylands phenology from 2001 to 2019, and their start of the growing season (SOS) in 2013 for (**b**) Australia and (**c**) southwestern North America drylands.

The improved phenology retrieval algorithm with dynamic pixel-wise amplitude threshold effectively improved the retrieval ratio (*RR*) of dryland phenology. The average *RR* of GDPD was 88.4% of global drylands over the period 2001–2019, outperforming other LSP products (Fig. 3a), which mainly benefited from the improved threshold^12^. The difference was also highlighted in several regions, such as the drylands of Australia and southwestern North America (Fig. 3b,c). In particular, MCD12Q2 retrieved phenology for only 39.5% of the global drylands on average, primarily due to its restrictive fixed amplitude threshold. Although AVH12 and VIPPHEN could successfully retrieve phenological events for most dryland regions, with average *RRs* of 81.9% and 81.6%, respectively, they may not accurately reflect the vegetation dynamics in heterogeneous dryland ecosystems due to coarse spatial resolution.

### Comparison of phenological metrics against PhenoCam GCC-retrieved results

**Fig. 4:**
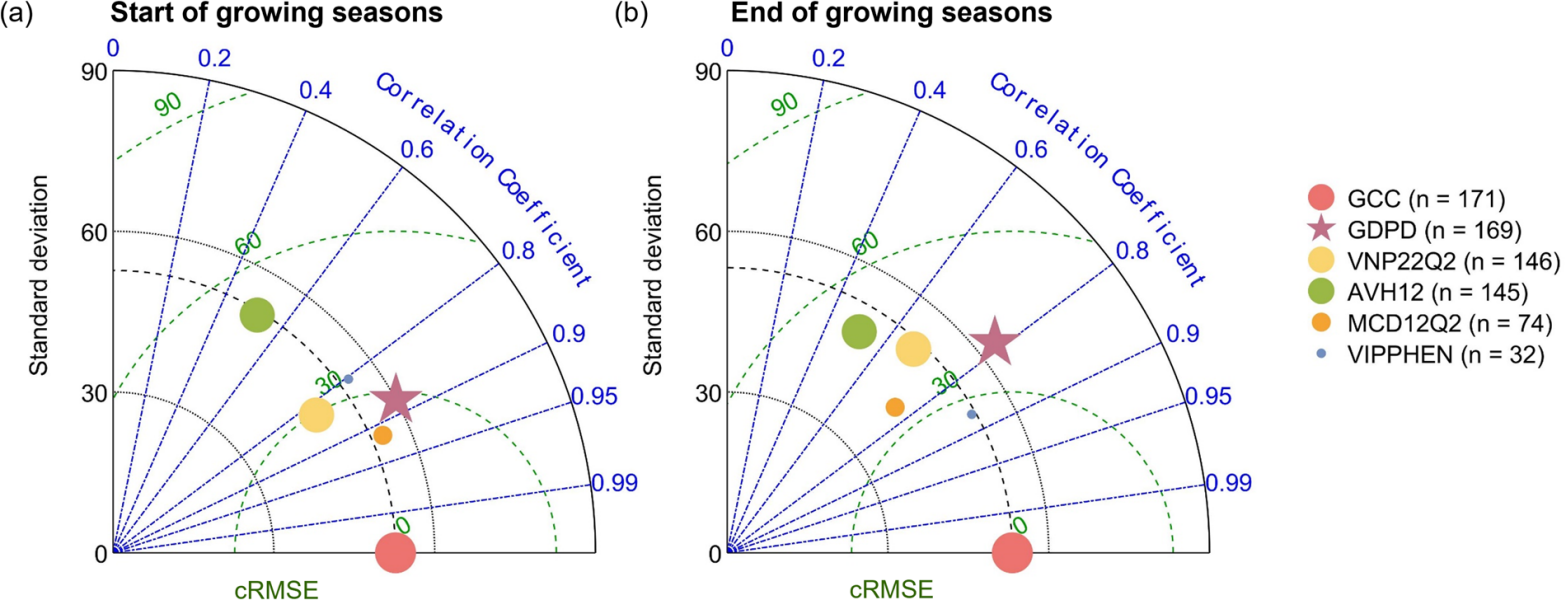
Taylor diagrams of (**a**) SOS and (**b**) EOS retrieved from GDPD, MCD12Q2, VNP22Q2, AVH12, VIPPHEN and PhenoCam sites. The red dot represents the PhenoCam observations, the star represents the GDPD. The scatter size represents the number of site-year pairs (n) matched between each LSP product and PhenoCam observations.

**Fig. 5:**
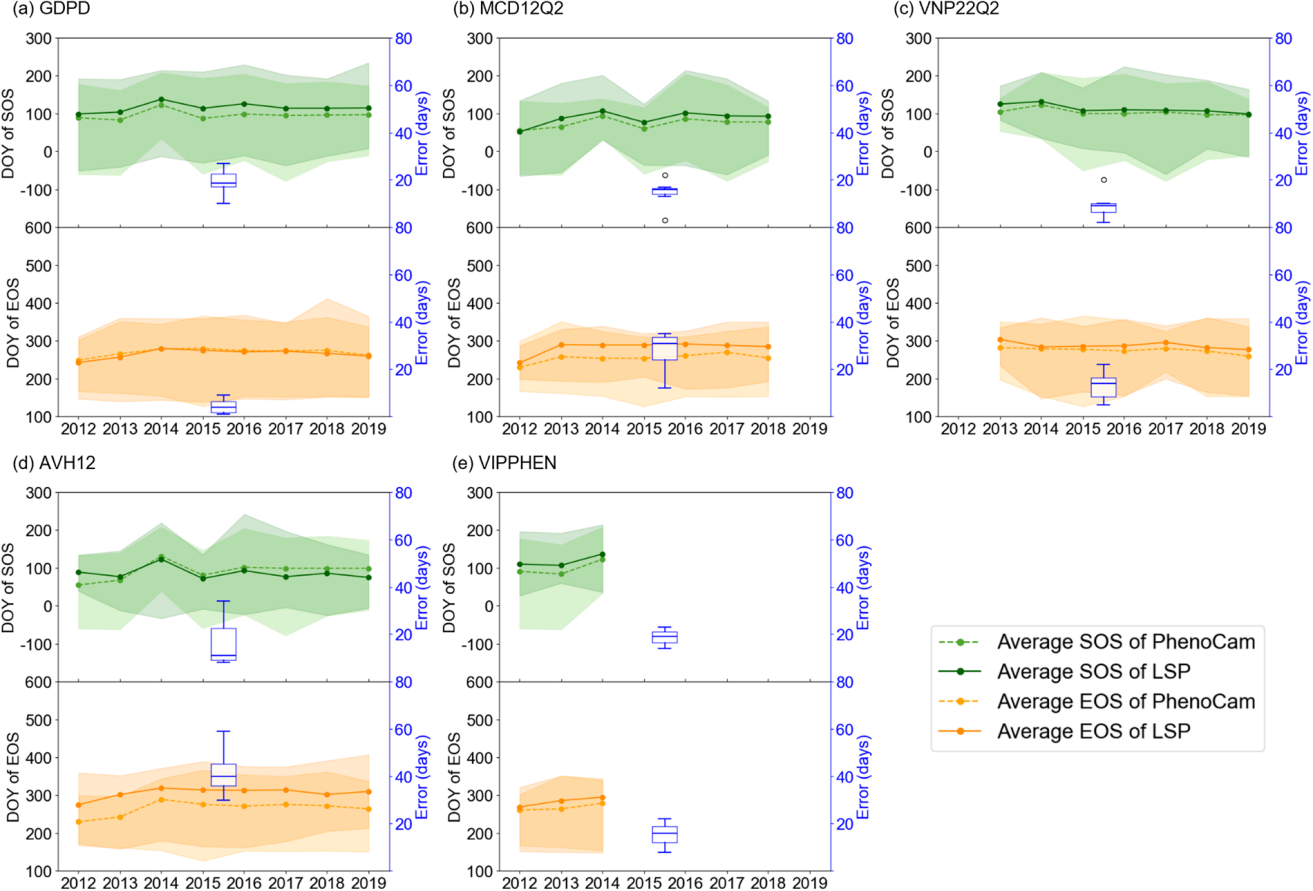
Comparison of the yearly average and variation of SOS and EOS at PhenoCam sites, using only sites available each year, between data from (**a**) GDPD, (**b**) MCD12Q2, (**c**) VNP22Q2, (**d**)AVH12, (**e**) VIPPHEN and GCC-derived SOS and EOS. Lines show the mean phenological metrics across available sites each year, with shaded areas representing the range between the earliest and latest values (i.e., minimum and maximum) among sites in each year. The blue boxplots (right y-axis) represent the average absolute error at PhenoCam sites across years where the horizontal lines represent the median values, “o” symbols indicate outliers. Gaps indicate years with missing data from LSP products.

Comparing GDPD and other LSP products (i.e., MCD12Q2, VNP22Q2, AVH12 and VIPPHEN) against the phenological metrics retrieved from PhenoCam GCC, GDPD presented better agreement with PhenoCam GCC-retrieved phenology in terms of phenological dates and spatial coverage (Fig. 4). For SOS, GDPD presented a high correlation (*r* = 0.88) and low bias (*cRMSE = *29 days) compared to GCC (Fig. 4a), outperforming all LSP products except MCD12Q2 (*r* = 0.92, *cRMSE = *23 days). However, MCD12Q2 showed much lower spatial coverage compared to GDPD, with only 74 samples matching the PhenoCam sites compared to 169 samples in GDPD. Notably, GDPD effectively captured the variation across sites and years (*SD* = 60 days) as indicated by PhenoCam GCC, which showed a strong agreement with the 1:1 line in the scatterplots (Fig. S2), suggesting the robustness of the improved phenology retrieval algorithm with dynamic pixel-wise amplitude threshold. Similarly, the EOS of GDPD also showed a higher correlation (*r* = 0.79) and a lower bias (*cRMSE = *39 days) with the PhenoCam observations compared to other LSP products, except for VIPPHEN and MCD12Q2 with low spatial coverage (Fig. 4b). Additionally, the GDPD presented the largest standard deviation for EOS (*SD* = 63 days) among all LSP products, which matched the range indicated by PhenoCam GCC, highlighting its ability to capture a wider range of EOS variations. Notably, all LSP products generally performed better for SOS than for EOS, which was also observed in regional dryland^30,46^ and non-dryland biomes^53,54^. It may be due to the fact that vegetation indices (e.g., EVI2 and NDVI) are primarily based on red and near-infrared reflectance to capture the change in greenness and vegetation health^55,56^. The other reason is the different viewing angles between the PhenoCam cameras and satellites^57^. The EOS starts with not only leaves browning but also leaves abscission^58^, the latter of which typically lags behind the color change during senescence^59^. Canopy gaps induced by leaf abscission are easily detected by the oblique views of ground-based digital cameras but not the top-of-canopy zenith views of satellite sensors due to canopy structural complexity^45^. In contrast, the rapid and distinct change of structure and pigment from senescence of the previous growing season to greening makes SOS easier to detect than EOS^60^. Nevertheless, GDPD effectively captured the interannual and spatial variability of EOS better than other LSP products (Figs. 5 and 7), providing an important reference for interpreting the effects of the vegetation senescence phase on carbon uptake.

Furthermore, to test the temporal robustness of GPDP across various spatial coverages, we calculated the year-specific averaged SOS and EOS where data were available for both the LSP product and PhenoCam sites, with the number of site-year pairs (*n*) used in each LSP product shown in Fig. 4. The absolute value of the error (right y-axis in Fig. 5) between the LSP products and the PhenoCam GCC-retrieved phenological metrics was used to evaluate the performance and stability of the phenological retrieval algorithms. All LSP products, including GDPD, presented similarly good agreement with the PhenoCam GCC-retrieved SOS (Fig. 5). For example, even though the site sets are different, all LSP products captured the widespread advancement in SOS in 2015 compared to 2014 showed in PhenoCam observations, except for VIPPHEN that lacked data after 2014. The GDPD algorithm also exhibited stable performance over all years, with an error of 19 ± 5 days for SOS (Fig. 5a), outperforming the AVH12, which had standard deviation of 9 days (Fig. 5d). Although MCD12Q2 (15 days, Fig. 5b) and VNP22Q2 (9 days, Fig. 5c) showed slightly lower average errors, this may be due to their lower spatiotemporal coverage (74 for MCD12Q2 and 146 for VNP22Q2). Both products also included notable outliers that inflated their overall variability. In contrast, GDPD exhibited the best performance for EOS retrieval among all LSP products, with the lowest error of 4 ± 3 days (Fig. 5a), closely matching the PhenoCam GCC-retrieved EOS. Moreover, GDPD more effectively captured the spatial variability of EOS indicated by PhenoCam (shaded area in Fig. 5) compared to MCD12Q2 and AVH12. The good performance in drylands was attributed to the fact that our algorithm prioritized dryland ecosystems, while other global LSP products did not. Nonetheless, the phenological metrics of all LSP products were generally later than those derived from the PhenoCam GCC.

### Comparison of phenological metrics against flux tower GPP-retrieved results

**Fig. 6:**
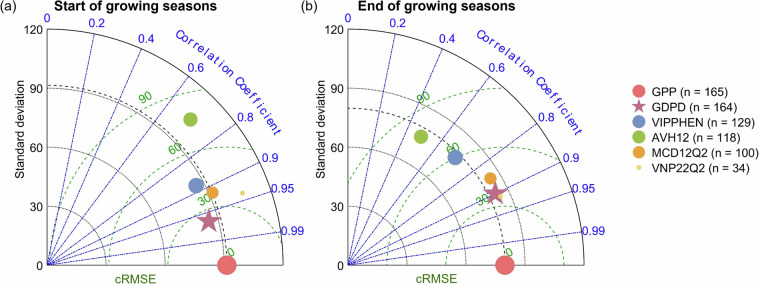
Taylor diagrams of (**a**) SOS and (**b**) EOS retrieved from GDPD, MCD12Q2, VNP22Q2, AVH12, VIPPHEN and flux tower GPP data. The red dot represents the flux tower observations, and the star represents the GDPD results. The scatter size represents the number of site-year pairs (n) matched between each LSP product and flux tower observations.

We further compared GDPD and other four LSP products (MCD12Q2, VNP22Q2, AVH12, and VIPPHEN) against the phenological metrics retrieved by daily GPP at flux tower sites. GDPD performed the best in matching flux tower observations, covering almost all flux tower sites (*n* = 164). Among LSP products with comparable spatial coverage, GDPD exhibited the highest correlation with both GPP-retrieved SOS (*r* = 0.97) and EOS (*r* = 0.90), along with the lowest bias (*cRMSEs* of 24 days for SOS and 37 days for EOS) (Fig. 6a). This indicated that GDPD was able to represent the onset and senescence of strong photosynthetic activity during the growing season. Moreover, the EOS of the GDPD exhibited the larger standard deviation (*SD* = 83 days), which better characterized the variation in EOS.

**Fig. 7:**
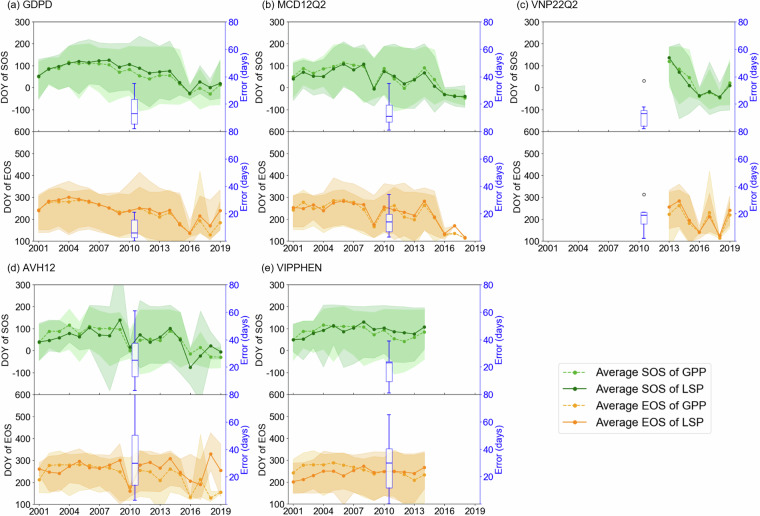
Comparison of the yearly average and variation of SOS and EOS at flux tower sites, using only sites available each year, between data from (**a**) GDPD, (**b**) MCD12Q2, (**c**) VNP22Q2, (**d**)AVH12, (**e**) VIPPHEN and GPP-derived SOS and EOS. Lines show the mean phenological metrics across available sites each year, with shaded areas representing the range between the earliest and latest values (i.e., minimum and maximum) among sites in each year. The blue boxplots (right y-axis) represent the average absolute error at flux tower sites across years where the horizontal lines represent the median values, “o” symbols represent outliers in the error distribution. Gaps indicate years with missing data from LSP products.

Similar to the evaluation with PhenoCam, we examined the temporal stability of retrieved phenological metrics from LSP products with the GPP-retrieved metrics (Fig. 7). For SOS, GDPD demonstrated high temporal consistency with identified in GPP-retrieved metrics, particularly in capturing similar temporal patterns observed in 2009 and 2016 (Fig. 7a). Among the evaluated LSP products, GDPD showed competitive performance in SOS estimation, with an error of 15 ± 10 days. Different from the evaluation with PhenoCam (Fig. 5d), AVH12 was difficult to accurately capture GPP-retrieved SOS, with an average error of 26 days, almost twice as great as other products (Fig. 7d). For EOS, GDPD showed the lowest temporal error (12 ± 14 days) compared to other LSP products. In addition, GDPD demonstrated strong consistency with the interannual variability of GPP-retrieved EOS. Notably, the year-to-year range of GDPD EOS across sites (shaded areas) closely aligns with that of the GPP data, particularly capturing early senescence events in dryland ecosystems. The better performance of GDPD than other LSP products, consistent with the evaluation with PhenoCam, was attributed to the fact that our algorithm prioritized dryland ecosystems, which other LSP products did not. Therefore, our dataset would facilitate future studies exploring the intrinsic link between canopy structure (represented by EVI2), pigmentation changes (represented by GCC), and photosynthesis (represented by GPP) in drylands.

### Impact of spatiotemporal resolutions of data source on phenological metrics

**Fig. 8:**
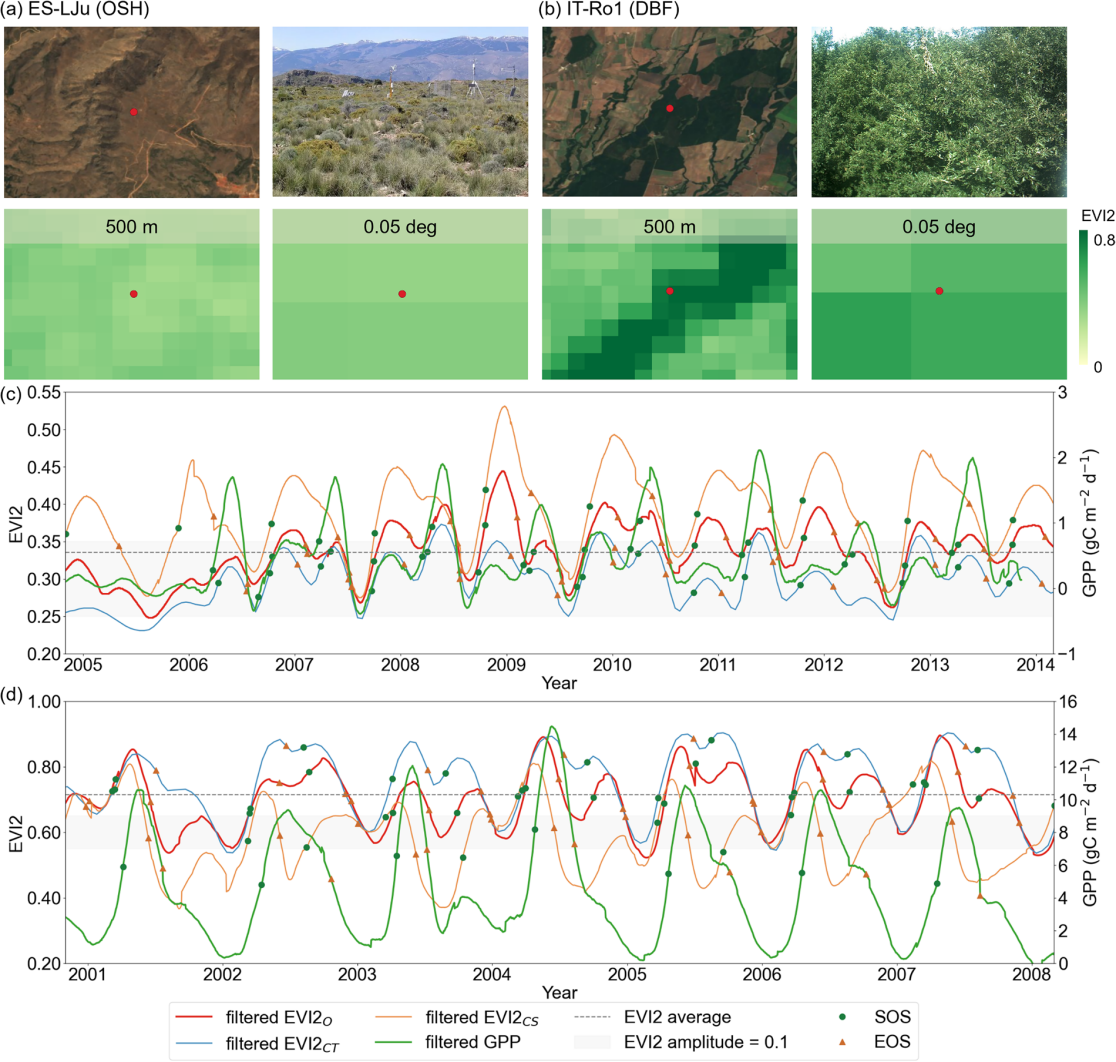
Time series of EVI2 at different spatial and temporal resolutions and daily GPP (green lines, right y-axis) at the (**a,c**) OSH (ES-LJu site) and (**b,d**) DBF (IT-Ro1 site) FLUXNET sites. EVI2_O_ (red lines), EVI2_CT_ (blue lines), and EVI2_CS_ (orange lines) represent the EVI2 time series with a spatial resolution of 500 m per day, spatial resolution of 500 m per 16 days, and spatial resolution of 0.05 deg per day, respectively. Green dots and orange triangles represent the beginning (SOS) and end (EOS) of the growing season.

To examine the influence of data sources with varying spatial and temporal resolutions on phenology retrieval, this study analyzed EVI2 time series at two spatial and two temporal scales and tested the retrieval algorithm at flux sites with two different vegetation types: ES-LJu with open shrubland (OSH) and IT-Ro1 with deciduous broadleaf forest (DBF). Here EVI2_O_ was adopted by GDPD using 500-m spatial resolution at the daily time step. It clearly showed that increasing the temporal steps to 16-day intervals (EVI2_CT_) led to smoother seasonal variations compared to EVI2_O_ at both flux sites, especially flattening the peak of the growing season. Consequently, SOS retrieved from EVI2_CT_ was generally earlier, while EOS tended to be later than those retrieved from EVI2_O_ (Fig. 8c). For instance, at the OSH site, EVI2_CT_-retrieved SOS was on average 13 days earlier and EOS was 2 days later than those from EVI2_O_. Similarly, at the DBF site, SOS occurred 3 days earlier and EOS 11 days later on average with EVI2_CT_. This is mainly because longer data intervals (i.e., coarser temporal resolutions) may skip or ignore the key phases of vegetation growth that are crucial for phenological metrics extraction, which was also shown in Tian *et al*.^61^.

In contrast, the effect of spatial resolution on phenological metrics varied depending on the local biomes. For example, at the OSH site with low vegetation coverage, finer spatial resolution data (500 m in EVI2_O_) successfully captured two growing seasons, while the second growing season was challenging to detect by coarser spatial resolution data (0.05 deg in EVI2_CS_) due to the mixed signals. In the other case of high vegetation coverage (DBF site), the second growing season was able to be detected by coarse resolution data for most years, but the difference of the EVI2 amplitude between fine and coarse resolution data was much larger for the second growing season than the first one. Considering such complexity due to heterogeneous ecosystems, finer resolution data are more appropriate for retrieving phenological metrics at the biome level or finer^37,62,63^.

Regarding physiological dynamics, EVI2_O_ matched better with the seasonal pattern and phenological date of the GPP at the OSH site (Fig. 8c) than data with lower temporal or lower spatial resolution. This highlighted the advantage of EVI2_O_ in accurately capturing seasonal carbon uptake dynamics^64^, which is critical for dryland biomes that respond rapidly to irregular changes in water availability. At the DBF site, the first growing season indicated by EVI2_O_ was consistent with GPP-retrieved phenological metrics, but the second growing season was barely shown by GPP. This discrepancy could be attributed to the fact that the flux tower GPP data primarily reflects phenological events of the dominant deciduous broadleaf forest. In contrast, the coarser-resolution satellite imagery may include mixed signals from surrounding vegetation types, such as grasses and shrubs (Fig. 8b). Consequently, the second growing season detected by EVI2_O_ may reflect greenness from nearby non-forest vegetation, rather than the actual phenology of the DBF.

## Usage Notes

GDPD provides high-accuracy and spatially extensive phenology information for global dryland regions, offering spatial coverage 2.2 times greater than that of MCD12Q2. However, there are still several limitations and directions for further investigation. Firstly, although the retrieval algorithm with dynamic, pixel-wise amplitude thresholds used by GDPD effectively improves the spatial extent of phenological retrievals, it may still fail to capture low-amplitude phenological events, such as during post-disturbance recovery or severe drought. Uncertainties in GPDP-derived phenological metrics are substantially higher in low-amplitude pixels, with SOS/EOS error SDs of 34/44 days (GCC) and 26/45 days, exceeding those in medium- and high-amplitude pixels (see Text S5, Figs. S6, S7). Future efforts could explore adaptive or context-aware thresholding strategies^41^ and spectral unmixing with more MOIDS reflectance bands^65^ to better extract vegetation dynamics out of background noises from bare soil, combustion, and other disturbing factors.

Secondly, there are mismatches between satellite-based LSP and *in-situ* observations due to the differences in observational scales and geometries, as well as the functional meaning of these metrics. On the one hand, discrepancies arise from differences in observational scale and sensor viewing geometry and depend on local biomes^22^. Although both EVI2 and GCC are sensitive to vegetation greenness, they differ in how this greenness is captured. GCC is derived from digital images taken in the visible spectrum with near-horizontal viewing angles, while satellite-based vegetation indices such as EVI2 rely on broader footprints, nadir-viewing geometries, and near-infrared reflectance. This mismatch is particularly pronounced in regions with low vegetation cover and heterogeneous landscapes (e.g., dryland shrub-grass-soil mosaics), where the seasonality of different biomes can be canceled out or aggregated within one pixel, resulting in large errors in retrieving phenological metrics than finer resolution data^23,37,63^. Scale limitations can be mitigated by using finer spatial resolution data, for example, using 30-m harmonized Landsat and Sentinel-2 (HLS) data, or even 3-m PlantScope images^30^ to accurately extract annual grasses^28^ and shrubs^10^ phenology in drylands. In addition, future research could use near-infrared-enabled digital cameras to calculate broadband (e.g., NDVI, EVI2) reflectance indices to match remote sensing-based phenological metrics^30,59^ and improve scalability as the network continues to expand.

On the other hand, mismatches are also driven by the fundamental decoupling between vegetation greenness and photosynthetic activity (represented by GPP). Theoretically, vegetation phenology is expected to be tightly coupled with photosynthetic processes^66^: climate warming often induces earlier green-up, which theoretically synchronizes with photosynthetic activation and promotes preferential carbon allocation to leaf development^67^. Photosynthesis in turn supplies essential substrates for vegetation growth, serving as the primary driver of canopy formation^68^. However, our results showed that GDPD-derived phenological metrics consistently lag behind *in-situ* observations, especially at the onset of the growing season (Figs. 5, 7). Similar lag pattern was also observed in other LSP products (Fig. 5) and previous studies^30,58,59,69^, suggesting a nonlinear relationship between canopy greenness and leaf physiology^58,70^. This decoupling may be due to differential responses of structural and physiological processes under stress, especially at specific growth stages^71^ and climatic conditions^72^. For example, drought-induced stomatal closure can significantly reduce photosynthetic activity even when canopy greenness remains high^72^. Such physiological lags are particularly common in dryland ecosystems^73^ and highlight the limitations of using greenness as a proxy for phenological activity. The observed delays in GDPD estimates are further supported by our evaluation results, where RMSE consistently exceeds cRMSE (Figs. S2, S3), indicating systematic bias in SOS retrievals linked to this greenness-photosynthesis decoupling. Recent advances in remote sensing technology offer new opportunities to better characterize vegetation physiological dynamics. In particular, solar-induced chlorophyll fluorescence (SIF) captures photosynthetic activity^74^ and can help bridge the gap between satellite-derived phenological metrics and the seasonal patterns of GPP observed at flux towers^75,76^.

Lastly, our results reveal that phenological retrievals in drylands exhibit greate variability and uncertainty across LSP products, highlighting the need for more *in-situ* observations and validations efforts in these regions. In particular, it is critical to expand field-based phenology monitoring in under-represented dryland areas, such as those in the Southern Hemisphere and high-latitude Northern Hemisphere^77^, to improve our understanding of vegetation dynamics across global drylands. Furthermore, existing *in-situ* datasets show an imbalanced representation of plant functional types (PFTs), with grasslands and shrublands dominating over other vegetation types (Tables S5, S6; Figs. S2, S3). This sampling bias may affect the robustness of model evaluation across all PFTs^78^. Therefore, establishing new validation sites in ecologically diverse and underrepresented PFTs will be essential to enhance the accuracy and generalizability of phenological models in dryland ecosystems.

Overall, GDPD is a dataset with high accuracy and spatial explicitly for dryland vegetation phenology, addressing critical gaps in existing dryland studies where low spatial coverage and coarse resolution satellite products (e.g., MCD12Q2) are limited in their ability to support a mechanistic understanding of vegetation-climate feedback in these fragile ecosystems. The enhanced spatiotemporal resolution of GDPD enables the detection of fine-scale phenological patterns across heterogeneous vegetation-soil mosaics, which is essential for capturing vegetation responses to climatic variability, land degradation, and disturbance regimes^79,80^. This capability is especially valuable for monitoring climate-sensitive species such as shrublands, which play a dominant role in the dryland carbon cycle but are often underrepresented in global LSP products^10^. Notably, the GDPD algorithm also demonstrates robustness across different satellite sensors (Text S6), supporting its applicability for future phenological monitoring as VIIRS increasingly replaces MODIS as the primary source of global optical remote sensing data. For future studies, GDPD can serve as a benchmark or assimilating dataset for the improvement of land surface models^21,81^, and inform the development of sustainable land use and resource management strategies^82^, including grazing practices^83^, restoration planning^84^, and early warning systems for drought and vegetation stress^85^.

## Supplementary information

Supplementary materials

## Data Availability

The Python code can be obtained through a public repository at https://github.com/ddd1207/GDPD.

## References

[1] Poulter, B. *et al*. Contribution of semi-arid ecosystems to interannual variability of the global carbon cycle. *Nature* **509**, 600–603 (2014). 24847888 10.1038/nature13376

[2] Ahlström, A. *et al*. The dominant role of semi-arid ecosystems in the trend and variability of the land CO _2_ sink. *Science* **348**, 895–899 (2015). 25999504 10.1126/science.aaa1668

[3] Yang, L. H. & Rudolf, V. H. W. Phenology, ontogeny and the effects of climate change on the timing of species interactions. *Ecol. Lett.* **13**, 1–10 (2010). 19930396 10.1111/j.1461-0248.2009.01402.x

[4] *Phenology and Seasonality Modeling*. **vol. 8** (Springer Berlin Heidelberg, Berlin, Heidelberg, 1974).

[5] Peñuelas, J., Rutishauser, T. & Filella, I. Phenology Feedbacks on Climate Change. *Science* **324**, 887–888 (2009). 19443770 10.1126/science.1173004

[6] Richardson, A. D. *et al*. Climate change, phenology, and phenological control of vegetation feedbacks to the climate system. *Agric. For. Meteorol.* **169**, 156–173 (2013).

[7] Zhang, X. *et al*. Monitoring vegetation phenology using MODIS. *Remote Sens. Environ.* **84**, 471–475 (2003).

[8] Caparros-Santiago, J. A., Rodriguez-Galiano, V. & Dash, J. Land surface phenology as indicator of global terrestrial ecosystem dynamics: A systematic review. *ISPRS J. Photogramm. Remote Sens.* **171**, 330–347 (2021).

[9] Zhang, X., Liu, L. & Yan, D. Comparisons of global land surface seasonality and phenology derived from AVHRR, MODIS, and VIIRS data. *J. Geophys. Res. Biogeosciences* **122**, 1506–1525 (2017).

[10] Peng, D. *et al*. Investigation of land surface phenology detections in shrublands using multiple scale satellite data. *Remote Sens. Environ.* **252**, 112133 (2021).

[11] Peng, D. *et al*. Intercomparison and evaluation of spring phenology products using National Phenology Network and AmeriFlux observations in the contiguous United States. *Agric. For. Meteorol.* **242**, 33–46 (2017).

[12] Xie, Q. *et al*. Land surface phenology retrievals for arid and semi-arid ecosystems. *ISPRS J. Photogramm. Remote Sens.* **185**, 129–145 (2022).

[13] Ye, Y. *et al*. An optimal method for validating satellite-derived land surface phenology using *in-situ* observations from national phenology networks. *ISPRS J. Photogramm. Remote Sens.* **194**, 74–90 (2022).

[14] Gray, J., Sulla-Menashe, D. & Friedl, M. A. User Guide to Collection 6.1 MODIS Land Cover Dynamics (MCD12Q2) Product. (2022).

[15] Broich, M. *et al*. A spatially explicit land surface phenology data product for science, monitoring and natural resources management applications. *Environ. Model. Softw.* **64**, 191–204 (2015).

[16] Ganguly, S., Friedl, M. A., Tan, B., Zhang, X. & Verma, M. Land surface phenology from MODIS: Characterization of the Collection 5 global land cover dynamics product. *Remote Sens. Environ.* **114**, 1805–1816 (2010).

[17] Taylor, S. D., Browning, D. M., Baca, R. A. & Gao, F. Constraints and Opportunities for Detecting Land Surface Phenology in Drylands. *J. Remote Sens.* **2021**, 2021/9859103 (2021).

[18] Gong, Z., Ge, W., Guo, J. & Liu, J. Satellite remote sensing of vegetation phenology: Progress, challenges, and opportunities. *ISPRS J. Photogramm. Remote Sens.* **217**, 149–164 (2024).

[19] Zhang, X. *et al*. Exploration of scaling effects on coarse resolution land surface phenology. *Remote Sens. Environ.* **190**, 318–330 (2017).

[20] Ma, X. *et al*. Spatial patterns and temporal dynamics in savanna vegetation phenology across the North Australian Tropical Transect. *Remote Sens. Environ.* **139**, 97–115 (2013).

[21] Smith, W. K. *et al*. Remote sensing of dryland ecosystem structure and function: Progress, challenges, and opportunities. *Remote Sens. Environ.* **233**, 111401 (2019).

[22] Peng, D. *et al*. Scaling up spring phenology derived from remote sensing images. *Agric. For. Meteorol.* **256–257**, 207–219 (2018).

[23] Liu, Y. *et al*. Using data from Landsat, MODIS, VIIRS and PhenoCams to monitor the phenology of California oak/grass savanna and open grassland across spatial scales. *Agric. For. Meteorol.* **237–238**, 311–325 (2017).

[24] Cheng, Y. *et al*. Phenology of short vegetation cycles in a Kenyan rangeland from PlanetScope and Sentinel-2. *Remote Sens. Environ.* **248**, 112004 (2020).

[25] Zhao, D. *et al*. Temporal resolution of vegetation indices and solar-induced chlorophyll fluorescence data affects the accuracy of vegetation phenology estimation: A study using *in-situ* measurements. *Ecol. Indic.* **136**, 108673 (2022).

[26] Zhang, X., Friedl, M. A. & Schaaf, C. B. Sensitivity of vegetation phenology detection to the temporal resolution of satellite data. *Int. J. Remote Sens.* **30**, 2061–2074 (2009).

[27] Wang, C. *et al*. Phenology Dynamics of Dryland Ecosystems Along the North Australian Tropical Transect Revealed by Satellite Solar-Induced Chlorophyll Fluorescence. *Geophys. Res. Lett.* **46**, 5294–5302 (2019).

[28] Pastick, N. J. *et al*. Characterizing Land Surface Phenology and Exotic Annual Grasses in Dryland Ecosystems Using Landsat and Sentinel-2 Data in Harmony. *Remote Sens.* **12**, 725 (2020).

[29] Leng, S. *et al*. Spatiotemporal Variations of Dryland Vegetation Phenology Revealed by Satellite-Observed Fluorescence and Greenness across the North Australian Tropical Transect. *Remote Sens.* **14**, 2985 (2022).

[30] Liu, Y. *et al*. Evaluation of PlanetScope-detected plant-specific phenology using infrared-enabled PhenoCam observations in semi-arid ecosystems. *ISPRS J. Photogramm. Remote Sens.* **210**, 242–259 (2024).

[31] UNEP-WCMC. A spatial analysis approach to the global delineation of dryland areas of relevance to the CBD Programme of Work on Dry and Subhumid Lands. Dataset based on spatial analysis between WWF terrestrial ecoregions and aridity zones. *UNEP-WCMC Resources* https://resources.unep-wcmc.org/products/https%3A%2F%2Fresources.unep-wcmc.org%2Fproducts%2F789fcac8959943ab9ed7a225e5316f08 (2007).

[32] Zomer, R. J. & Trabucco, A. Version 3 of the “Global Aridity Index and Potential Evapotranspiration (ET0) Database”: Estimation of Penman-Monteith Reference Evapotranspiration. https://csidotinfo.wordpress.com/2019/01/24/global-aridity-index-and-potential-evapotranspiration-climate-database-v3/ (2022). 10.1038/s41597-022-01493-1 PMC9287331 35840601

[33] Friedl, M. & Sulla-Menashe, D. MODIS/Terra+Aqua Land Cover Type Yearly L3 Global 500m SIN Grid V061 [Data set]. 10.5067/MODIS/MCD12Q1.061 (2022).

[34] Schaaf, C. & Wang, Z. MODIS/Terra+Aqua BRDF/Albedo Nadir BRDF Adjusted Ref Daily L3 Global - 500m V061 [Data set]. 10.5067/MODIS/MCD43A4.061 (2021).

[35] Ma, X., Huete, A. & Tran, N. N. Interaction of Seasonal Sun-Angle and Savanna Phenology Observed and Modelled using MODIS. *Remote Sens.* **11**, 1398 (2019).

[36] Jiang, Z., Huete, A. R., Didan, K. & Miura, T. Development of a two-band enhanced vegetation index without a blue band. *Remote Sens. Environ.* **112**, 3833–3845 (2008).

[37] Klosterman, S. T. *et al*. Evaluating remote sensing of deciduous forest phenology at multiple spatial scales using PhenoCam imagery. *Biogeosciences* **11**, 4305–4320 (2014).

[38] Didan, K. & Barreto, A. NASA MEaSUREs Vegetation Index and Phenology (VIP) Phenology EVI2 Yearly Global 0.05Deg CMG [Data set]. 10.5067/MEaSUREs/VIP/VIPPHEN_EVI2.004 (2024).

[39] Chen, J. *et al*. A simple method for reconstructing a high-quality NDVI time-series data set based on the Savitzky–Golay filter. *Remote Sens. Environ.* **91**, 332–344 (2004).

[40] Moon, M., Richardson, A. D., Milliman, T. & Friedl, M. A. A high spatial resolution land surface phenology dataset for AmeriFlux and NEON sites. *Sci. Data* **9**, 448 (2022). 35896546 10.1038/s41597-022-01570-5 PMC9329431

[41] Xie, Q. *et al*. Land surface phenology indicators retrieved across diverse ecosystems using a modified threshold algorithm. *Ecol. Indic.* **147**, 110000 (2023).

[42] Cañete, E. P. S. *et al*. FLUXNET2015 ES-LJu Llano de los Juanes. FluxNet; University of Granada 10.18140/FLX/1440157 (2016).

[43] Valentini, R. *et al*. FLUXNET2015 IT-Ro1 Roccarespampani 1. FluxNet; University of Tuscia - Vietrbo 10.18140/FLX/1440174 (2016).

[44] Seyednasrollah, B. *et al*. PhenoCam Dataset v2.0: Vegetation Phenology from Digital Camera Imagery, 2000–2018. 10.3334/ORNLDAAC/1674 (2019).

[45] D’Odorico, P. *et al*. The match and mismatch between photosynthesis and land surface phenology of deciduous forests. *Agric. For. Meteorol.* **214–215**, 25–38 (2015).

[46] Zhang, X. *et al*. Evaluation of land surface phenology from VIIRS data using time series of PhenoCam imagery. *Agric. For. Meteorol.* **256–257**, 137–149 (2018).

[47] Sonnentag, O. *et al*. Digital repeat photography for phenological research in forest ecosystems. *Agric. For. Meteorol.* **152**, 159–177 (2012).

[48] Browning, D. M., Karl, J. W., Morin, D., Richardson, A. D. & Tweedie, C. E. Phenocams Bridge the Gap between Field and Satellite Observations in an Arid Grassland Ecosystem. *Remote Sens.* **9**, 1071 (2017).

[49] Tian, F. *et al*. Calibrating vegetation phenology from Sentinel-2 using eddy covariance, PhenoCam, and PEP725 networks across Europe. *Remote Sens. Environ.* **260**, 112456 (2021).

[50] Wang, X. *et al*. No trends in spring and autumn phenology during the global warming hiatus. *Nat. Commun.* **10**, 1–10 (2019). 31160586 10.1038/s41467-019-10235-8 PMC6546754

[51] Taylor, K. E. Summarizing multiple aspects of model performance in a single diagram. *J. Geophys. Res. Atmospheres* **106**, 7183–7192 (2001).

[52] Dong, Y. *et al*. Global dryland phenology dataset (GDPD). 20976713798 Bytes figshare 10.6084/m9.figshare.27160602.v2 (2024).

[53] Wu, C. *et al*. Land surface phenology derived from normalized difference vegetation index (NDVI) at global FLUXNET sites. *Agric. For. Meteorol.* **233**, 171–182 (2017).

[54] Zhang, C., Zhang, Y., Wang, Z., Li, J. & Odeh, I. Monitoring Phenology in the Temperate Grasslands of China from 1982 to 2015 and Its Relation to Net Primary Productivity. *Sustainability* **12**, 12 (2020).

[55] Xue, J. & Su, B. Significant Remote Sensing Vegetation Indices: A Review of Developments and Applications. *J. Sens.* **2017**, 1–17 (2017).

[56] Motohka, T., Nasahara, K. N., Oguma, H. & Tsuchida, S. Applicability of Green-Red Vegetation Index for Remote Sensing of Vegetation Phenology. *Remote Sens.* **2**, 2369–2387 (2010).

[57] Liu, Y. *et al*. Modeling plant phenology by MODIS derived photochemical reflectance index (PRI). *Agric. For. Meteorol.* **324**, 109095 (2022).

[58] Keenan, T. F. *et al*. Tracking forest phenology and seasonal physiology using digital repeat photography: a critical assessment. *Ecol. Appl.* **24**, 1478–1489 (2014). 29160668 10.1890/13-0652.1

[59] Filippa, G. *et al*. NDVI derived from near-infrared-enabled digital cameras: Applicability across different plant functional types. *Agric. For. Meteorol.* **249**, 275–285 (2018).

[60] Gallinat, A. S., Primack, R. B. & Wagner, D. L. Autumn, the neglected season in climate change research. *Trends Ecol. Evol.* **30**, 169–176 (2015). 25662784 10.1016/j.tree.2015.01.004

[61] Tian, J., Zhu, X., Wan, L. & Collin, M. Impacts of Satellite Revisit Frequency on Spring Phenology Monitoring of Deciduous Broad-Leaved Forests Based on Vegetation Index Time Series. *IEEE J. Sel. Top. Appl. Earth Obs. Remote Sens.* **14**, 10500–10508 (2021).

[62] Peng, D. *et al*. Scaling effects on spring phenology detections from MODIS data at multiple spatial resolutions over the contiguous United States. *ISPRS J. Photogramm. Remote Sens.* **132**, 185–198 (2017).

[63] Xu, K. *et al*. How Spatial Resolution Affects Forest Phenology and Tree-Species Classification Based on Satellite and Up-Scaled Time-Series Images. *Remote Sens.* **13**, 2716 (2021).

[64] Cui, T., Martz, L., Zhao, L. & Guo, X. Investigating the impact of the temporal resolution of MODIS data on measured phenology in the prairie grasslands. *GIScience Remote Sens.* **57**, 395–410 (2020).

[65] Nagol, J. R., Sexton, J. O., Anand, A., Sahajpal, R. & Edwards, T. C. Isolating type-specific phenologies through spectral unmixing of satellite time series. *Int. J. Digit. Earth* **11**, 233–245 (2018).

[66] Fang, J. *et al*. Optimal representation of spring phenology on photosynthetic productivity across the northern hemisphere forests. *Agric. For. Meteorol.* **350**, 109975 (2024).

[67] Gao, S. *et al*. An earlier start of the thermal growing season enhances tree growth in cold humid areas but not in dry areas. *Nat. Ecol. Evol.* **6**, 397–404 (2022). 35228669 10.1038/s41559-022-01668-4

[68] Paul, M. J. & Pellny, T. K. Carbon metabolite feedback regulation of leaf photosynthesis and development. *J. Exp. Bot.* **54**, 539–547 (2003). 12508065 10.1093/jxb/erg052

[69] Yang, X., Tang, J. & Mustard, J. F. Beyond leaf color: Comparing camera-based phenological metrics with leaf biochemical, biophysical, and spectral properties throughout the growing season of a temperate deciduous forest. *J. Geophys. Res. Biogeosciences* **119**, 181–191 (2014).

[70] Brown, L. A., Dash, J., Ogutu, B. O. & Richardson, A. D. On the relationship between continuous measures of canopy greenness derived using near-surface remote sensing and satellite-derived vegetation products. *Agric. For. Meteorol.* **247**, 280–292 (2017).

[71] Ma, X. *et al*. Monitoring nature’s calendar from space: Emerging topics in land surface phenology and associated opportunities for science applications. *Glob. Change Biol.* **28**, 7186–7204 (2022). 10.1111/gcb.16436 PMC9827868 36114727

[72] Yang, J. *et al*. Amazon drought and forest response: Largely reduced forest photosynthesis but slightly increased canopy greenness during the extreme drought of 2015/2016. *Glob. Change Biol.* **24**, 1919–1934 (2018). 10.1111/gcb.14056 29345031

[73] Wang, X. *et al*. Globally Consistent Patterns of Asynchrony in Vegetation Phenology Derived From Optical, Microwave, and Fluorescence Satellite Data. *J. Geophys. Res. Biogeosciences* **125**, e2020JG005732 (2020).

[74] Walther, S. *et al*. Satellite chlorophyll fluorescence measurements reveal large-scale decoupling of photosynthesis and greenness dynamics in boreal evergreen forests. *Glob. Change Biol.* **22**, 2979–2996 (2016). 10.1111/gcb.13200 26683113

[75] Chen, A. *et al*. Seasonal changes in GPP/SIF ratios and their climatic determinants across the Northern Hemisphere. *Glob. Change Biol.* **27**, 5186–5197 (2021). 10.1111/gcb.15775 34185345

[76] Zhang, J. *et al*. Solar-induced chlorophyll fluorescence captures photosynthetic phenology better than traditional vegetation indices. *ISPRS J. Photogramm. Remote Sens.* **203**, 183–198 (2023).

[77] Brown, T. B. *et al*. Using phenocams to monitor our changing Earth: toward a global phenocam network. *Front. Ecol. Environ.* **14**, 84–93 (2016).

[78] Jose, K., Chaturvedi, R. K., Jeganathan, C., Behera, M. D. & Singh, C. P. Plugging the Gaps in the Global PhenoCam Monitoring of Forests—The Need for a PhenoCam Network across Indian Forests. *Remote Sens.* **15**, 5642 (2023).

[79] Tagesson, T. *et al*. Spatiotemporal variability in carbon exchange fluxes across the Sahel. *Agric. For. Meteorol.* **226–227**, 108–118 (2016).

[80] Bernardino, P. N. *et al*. Uncovering Dryland Woody Dynamics Using Optical, Microwave, and Field Data—Prolonged Above-Average Rainfall Paradoxically Contributes to Woody Plant Die-Off in the Western Sahel. *Remote Sens.* **12**, 2332 (2020).

[81] Renwick, K. M. *et al*. Modeling phenological controls on carbon dynamics in dryland sagebrush ecosystems. *Agric. For. Meteorol.* **274**, 85–94 (2019).

[82] Walker, J. J. & Soulard, C. E. Phenology Patterns Indicate Recovery Trajectories of Ponderosa Pine Forests After High-Severity Fires. *Remote Sens.* **11**, 2782 (2019).

[83] Tanda, A. *et al*. Land surface phenology for the characterization of Mediterranean permanent grasslands. *Precis. Agric.* **26**, 16 (2024).

[84] Buisson, E., Alvarado, S. T., Stradic, S. L. & Morellato, L. P. C. Plant phenological research enhances ecological restoration. *Restor. Ecol.* **25**, 164–171 (2017).

[85] Delalieux, S. *et al*. Hyperspectral indices to diagnose leaf biotic stress of apple plants, considering leaf phenology. *Int. J. Remote Sens.* **30**, 1887–1912 (2009).

[86] Friedl, M., Gray, J. & Sulla-Menashe, D. MODIS/Terra+Aqua Land Cover Dynamics Yearly L3 Global 500m SIN Grid V061 [Data set]. 10.5067/MODIS/MCD12Q2.061 (2022).

[87] Zhang, X. *et al*. Generation and evaluation of the VIIRS land surface phenology product. *Remote Sens. Environ.* **216**, 212–229 (2018).

[88] Wu, W., Sun, Y., Xiao, K. & Xin, Q. Development of a global annual land surface phenology dataset for 1982–2018 from the AVHRR data by implementing multiple phenology retrieving methods. *Int. J. Appl. Earth Obs. Geoinformation* **103**, 102487 (2021).

[89] Cao, R., Chen, J., Shen, M. & Tang, Y. An improved logistic method for detecting spring vegetation phenology in grasslands from MODIS EVI time-series data. *Agric. For. Meteorol.* **200**, 9–20 (2015).

