# Supplementary material for: An Enhanced Phenology Dataset for Global Drylands from 2001 to 2019
Source: Sci Data. 2025 Jul 9;12:1167. doi: 10.1038/s41597-025-05519-2 (PMC12241349; doi:10.1038/s41597-025-05519-2)
Supplement: Supplementary file 1 — Supplementary materials [file 41597_2025_5519_MOESM1_ESM.pdf]

Supplementary materials

Text S1. Quality control for selected LSP products

The MCD12Q2 product provides detailed quality assurance (detailed QA) data for each phenological metric, and for the entire growing season (overall QA)[1]. The QA values range from 0 to 3, corresponding to "best", "good", "fair", and "poor". In this study, we retained pixels for which the overall QA was best or good and detailed QA for greenup and senescence as best or good.

The VNP22Q2 product also provides quality control (GLSP_QC) at each pixel based on overall quality assurance[2]. We selected pixels with a mandatory quality of 0 per year, which indicated "processed, high quality".

The AVH12 product provides the amplitude threshold (AT), the first-order derivative method (FOD), the second-order derivative method (SOD), relative changing rate method (RCR), the third-order derivative method (TOD) and the curvature change rate method (CCR) six methods to retrieve SOS and EOS[3]. In this study, we chose the AT method, consistent with GDPD retrieval method. However, AVH12 does not provide reliability layers, so we did not perform quality control for it.

The VIPPHEN product provides the phenology reliability layer[4], and we choose pixels with QA = 0 or 1 to indicate "excellent" and "good" reliability, respectively.

Text S2. Retrieving phenology metrics from flux tower GPP data with different amplitude thresholds

Tian et al.[5] calculated GPP at varying thresholds from 5% to 50% by a step of 5%, which were used as references for analyzing how well the VI-derived SOS/EOS reflects the spatial variability in the GPP-derived SOS/EOS across the 52 sites in Europe. The results revealed a better correlation and less bias with the GPP-derived SOS/EOS extracted from 20%, 30% and 50% thresholds at 50% EVI2 thresholds. We therefore adopted this strategy and calculated the correlation between GPP-derived SOS/EOS extracted at 20%, 30%, and 50% thresholds and EVI2-derived SOS/EOS extracted at 50% thresholds at the flux tower sites in the drylands respectively. We found that the GPP amplitude threshold with 20% has the best correlation with the SOS (r = 0.97) and EOS (r = 0.90) retrieved from EVI2 (Fig. S1), so all subsequent validations were using the 20% threshold.

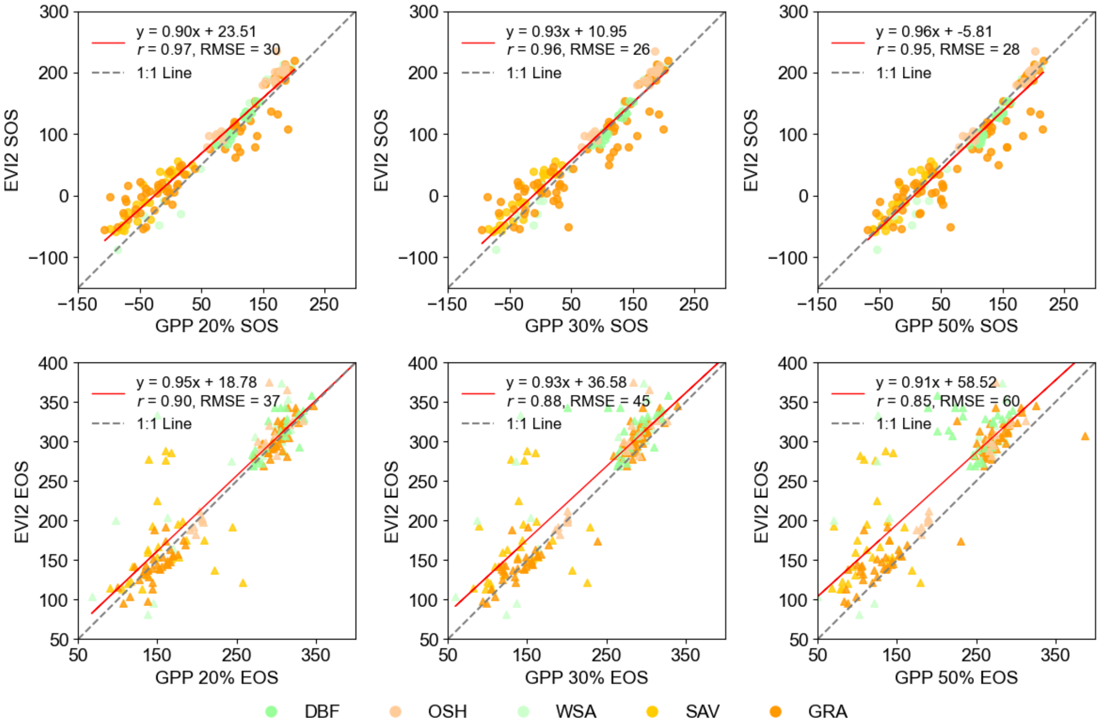

Figure S1. Scatterplots of SOS (circles) / EOS (triangles) derived from EVI2 at 50% seasonal amplitude threshold versus those derived from GPP at 20%, 30%, and 50% thresholds. The red solid line is the linear regression line and the gray dashed line is the 1:1 line. *r* is the Pearson correlation coefficient value, and RMSE is the root mean square error.

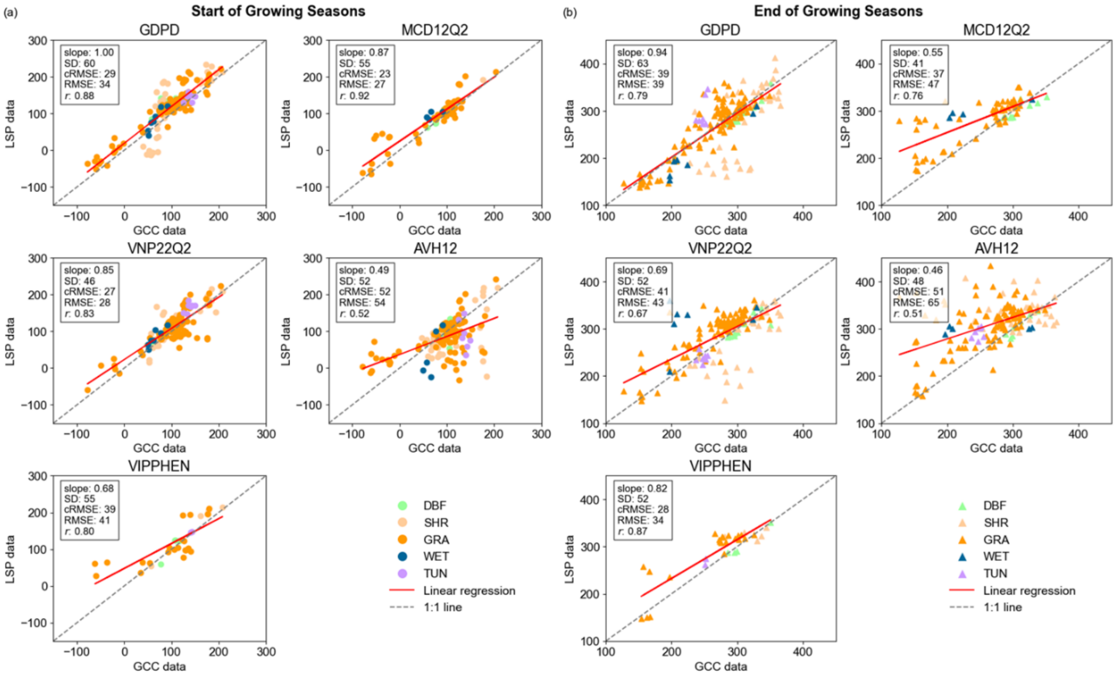

Figure S2. Scatterplots and linear regression of (a) SOS and (b) EOS from GDPD, MCD12Q2, VNP22Q2,

AVH12, VIPPHEN and PhenoCam observations. The red solid line is the linear regression line and the gray dashed line is the 1:1 line. SD, cRMSE, RMSE and *r* represent the standard deviation, centered root-mean-square error, root mean square error and correlation coefficient of the LSP product respectively.

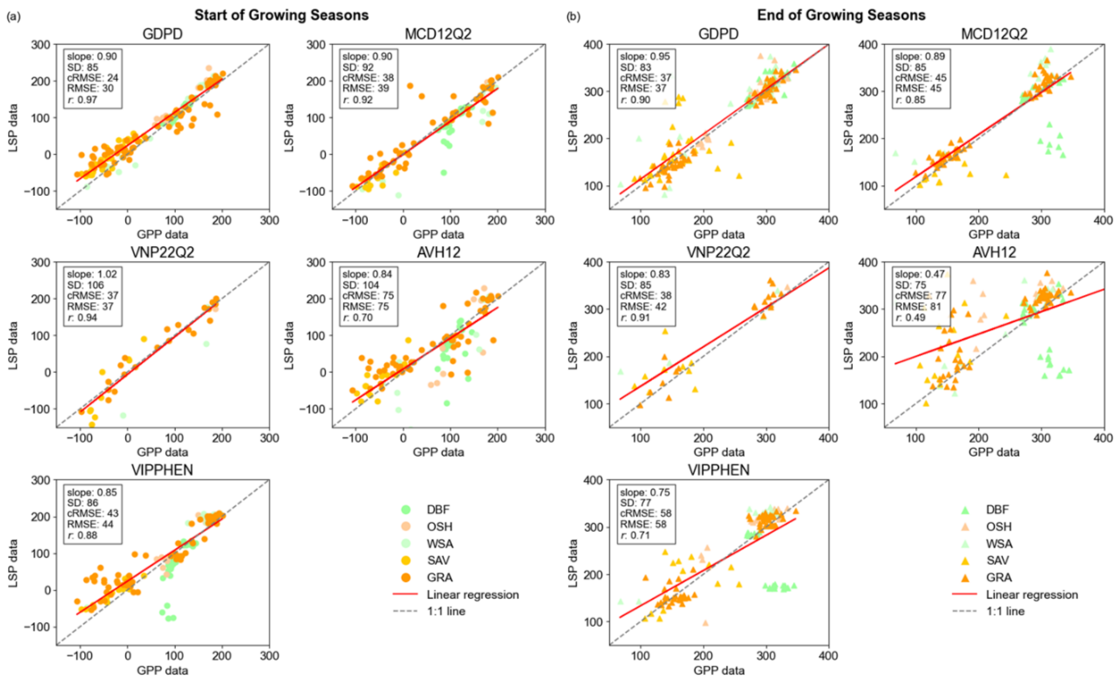

Figure S3. Scatterplots and linear regression of (a) SOS and (b) EOS from GDPD, MCD12Q2, VNP22Q2, AVH12, VIPPHEN and flux tower observations. The red solid line is the linear regression line and the gray dashed line is the 1:1 line. SD, cRMSE, RMSE and *r* represent the standard deviation, centered root-mean-square error, root mean square error and correlation coefficient of the LSP product respectively.

Table S1. Correlation coefficients between phenological metrics of LSP products and PhenoCam observations for various vegetation types.

		GDPD	MCD12Q2	VNP22Q2	AVH12	VIPPHEN
SOS	DBF	0.67	0.98	0.99	0.49	0.97
	SHR	0.80	–	0.85	0.54	0.98
	GRA	0.94	0.91	0.81	0.54	0.74
	WET	0.94	0.67	0.90	0.85	–
	TUN	0.20	–	0.55	–0.58	–
EOS	DBF	0.95	0.87	0.78	0.94	0.98
	SHR	0.70	–	0.73	–0.01	0.29

	GRA	0.90	0.77	0.86	0.53	0.88
	WET	0.98	0.97	0.29	0.35	–
	TUN	0.60	–	–0.03	0.29	–

Table S2. Correlation coefficients between phenological metrics of LSP products and flux tower observations for various vegetation types.

		GDPD	MCD12Q2	VNP22Q2	AVH12	VIPPHEN
SOS	DBF	0.96	0.72	–	0.04	0.75
	OSH	0.98	–	–	0.77	0.95
	WSA	0.99	0.94	0.95	0.72	0.98
	SAV	0.93	0.78	0.94	0.81	0.94
	GRA	0.95	0.92	0.97	0.86	0.93
EOS	DBF	0.79	–0.27	–	–0.46	–0.88
	OSH	0.93	–	–	0.57	0.86
	WSA	0.81	0.97	0.96	0.68	0.98
	SAV	0.17	0.29	0.21	0.39	0.46
	GRA	0.97	0.98	0.93	0.70	0.97

Text S3. Comparison of phenological metrics against consistently retrieved site-years from PhenoCam GCC- and flux tower GPP-retrieved results

To ensure a fair comparison among the different LSP products, we only compared site-year pairs that were successfully retrieved across all products.

For PhenoCam sites, there were 9 matched site-year pairs across all LSP products. Among all LSP products, GDPD demonstrated superior performance (Fig. S4), with the highest correlation with PhenoCam observations (SOS: $r = 0.91$, EOS: $r = 0.98$) and the lowest bias (SOS: cRMSE = 17 days, EOS: cRMSE = 13 days).

For the flux sites, 8 site-year pairs were available across all products (Fig. S5). In terms of SOS, GDPD showed comparable accuracy to AVH12 and VIPPHEN, with a correlation coefficient of $r = 0.95$ and cRMSE of 25 days. For EOS, GDPD outperformed other products, with a correlation greater than $r > 0.99$ and cRMSE = 10 days.

This analysis further supports the robustness and accuracy of the improved phenology retrieval algorithm, particularly when evaluated under consistent conditions across methods.

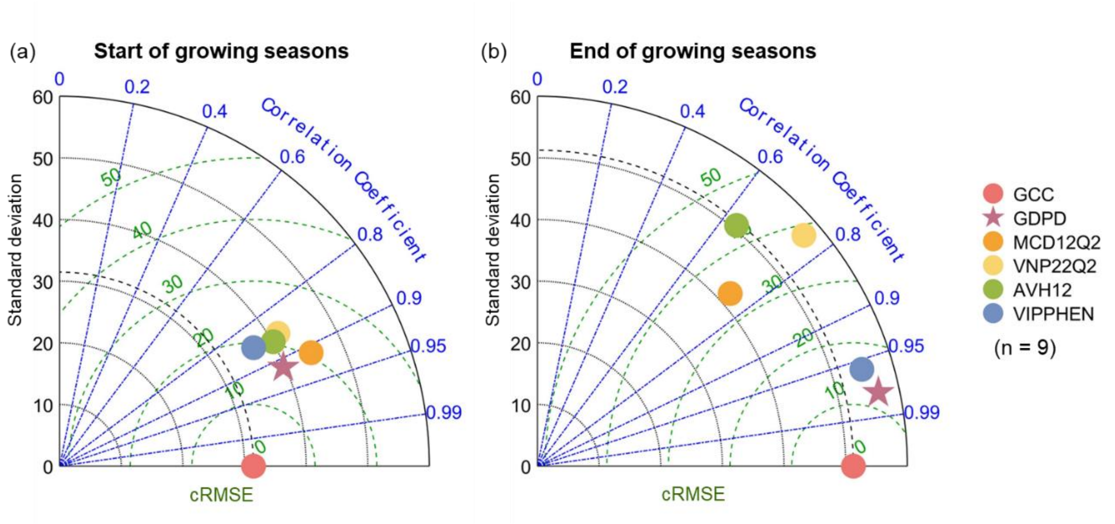

Figure S4. Taylor diagrams of (a) SOS and (b) EOS retrieved from GDPD, MCD12Q2, VNP22Q2, AVH12, VIPPHEN and PhenoCam sites where all LSP products successfully retrieved phenological metrics. The red dot represents the PhenoCam observations, the star represents the GDPD.

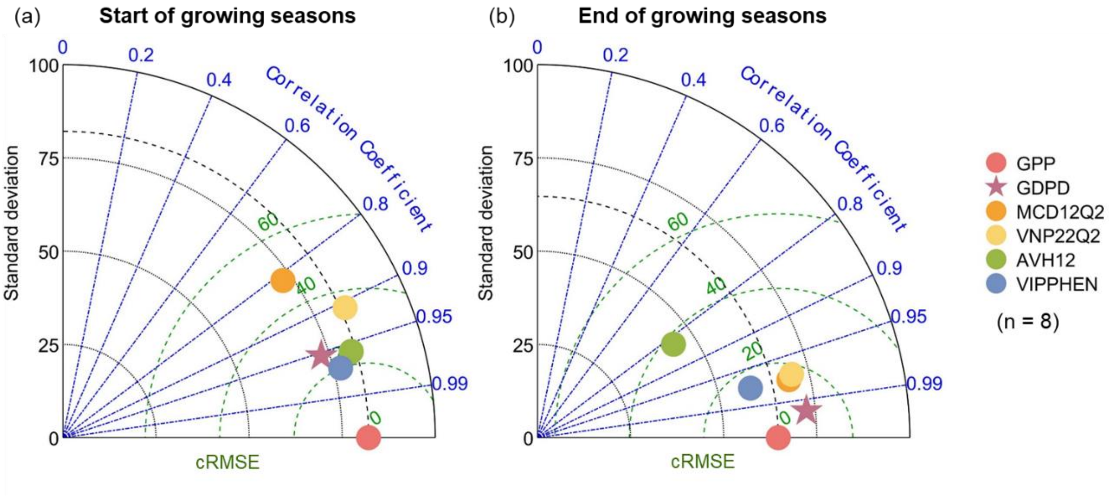

Figure S5. Taylor diagrams of (a) SOS and (b) EOS retrieved from GDPD, MCD12Q2, VNP22Q2, AVH12, VIPPHEN and flux tower data where all LSP products successfully retrieved phenological metrics. The red dot represents the flux tower observations, and the star represents the GDPD results.

Text S4. Comparison of phenological metrics of the second growing season against PhenoCam GCC- and flux tower GPP-retrieved results

First, we extracted phenological metrics for PhenoCam and flux tower sites that exhibited two growing seasons. Second, we extracted the corresponding phenological metrics of the second growing season

from GDPD and other LSP products. Of these, the MCD12Q2, VNP22Q2 and VIPPHEN products provided phenological metrics of the second growing season, while the AVH12 products did not, therefore it was not evaluated.

For PhenoCam sites, only freemangrass site retrieved two growing seasons in 2012, and GDPD showed high consistency both at the start and end of the second growing season (SOS2 and EOS2) (Table S3). However, VIPPHEN had poorer consistency, MCD12Q2 and VNP22Q2 failed to retrieve the second growing season.

For the flux sites, four sites retrieved two growing seasons in ten years, with GDPD retrieving 50% correspondingly, outperforming other LSP products (Table S4). Similar to the PhenoCam evaluation, GDPD exhibited the highest consistency with GPP-retrieved SOS of the second growing season (r = 0.99), with the lowest bias (*cRMSEs* = 7 days). Although the EOS of the second growing season retrieved by GDPD was less consistent with GPP-retrieved results (r = 0.42), it still outperformed VIPPHEN (Table S4). Both MCD12Q2 and VNP22Q2 failed to retrieve the second growing season across these flux sites.

Table S3. Phenological metrics of the second growing season retrieved from PhenoCam, GDPD, MCD12Q2, VIPPHEN and VNP22Q2. SOS2 is the start of the second growing season and EOS2 is the end of the second growing season.

Site name	Year	SOS2					EOS2				
		PhenoCam GCC	GDPD	MCD12Q2	VIPPHEN	VNP22Q2	PhenoCam GCC	GDPD	MCD12Q2	VIPPHEN	VNP22Q2
freemangrass	2012	225	229	/	261	/	358	355	/	349	/

Table S4. Phenological metrics of the second growing season retrieved from flux tower, GDPD, MCD12Q2, VIPPHEN and VNP22Q2. SOS2 is the start of the second growing season and EOS2 is the end of the second growing season.

Site name	Year	SOS2					EOS2				
		Flux tower GPP	GDPD	MCD12Q2	VIPPHEN	VNP22Q2	Flux tower GPP	GDPD	MCD12Q2	VIPPHEN	VNP22Q2
AU-ASM	2010	203	234	/	/	/	320	373	/	/	/
AU-Cum	2016	209	/	/	/	/	369	/	/	/	/
AU-Cum	2017	205	/	/	/	/	381	/	/	/	/
AU-Cum	2019	223	/	/	/	/	370	/	/	/	/
AU-GWW	2014	233	/	/	/	/	382	/	/	/	/
AU-GWW	2018	256	/	/	/	/	392	/	/	/	/
ES-LJu	2006	240	262	/	97	/	409	380	/	261	/
ES-LJu	2007	247	261	/	/	/	392	405	/	/	/

ES-LJu	2009	243	260	/	/	/	368	402	/	/	/
ES-LJu	2012	258	269	/	/	/	381	393	/	/	/

Text S5. Relationship between phenological retrieval accuracy and seasonal amplitude

To investigate the potential relationship between the accuracy of phenological retrieval and seasonal vegetation amplitude, we performed a correlation analysis between them. We quantified the errors of GDPD SOS and EOS relative to ground observations (PhenoCam GCC and flux tower GPP data) and examined their linear correlations with corresponding seasonal amplitudes across all available site-years.

The analysis revealed no significant relationship between SOS error and seasonal amplitude at either PhenoCam sites (Fig. S6a) or flux tower sites (Fig. S7a). For EOS error, we observed statistically significant but weak correlations: $r = 0.24$ ($p < 0.05$) for PhenoCam sites (Fig. S6b) and $r = -0.18$ ($p < 0.05$) for flux tower sites (Fig. S7b). However, these correlations should be interpreted cautiously given: (1) the limited sample size in high-amplitude ranges, and (2) potential influence of outliers on linear regression results.

To provide more robust insights, we classified seasonal amplitudes into three groups: low (< 0.3), medium (0.3–0.6), and high (≥ 0.6), then compared SOS/EOS errors across these groups. A comprehensive comparison of phenological retrieval errors across these groups was conducted using one-way ANOVA followed by Tukey's HSD post-hoc test. The statistical analysis revealed no significant differences in retrieval errors among the three amplitude groups (ANOVA: $p > 0.05$ for both SOS and EOS; Tukey HSD: all pairwise comparisons $p > 0.05$). These results confirm that the accuracy of GDPD phenological metrics remains largely independent of seasonal amplitudes.

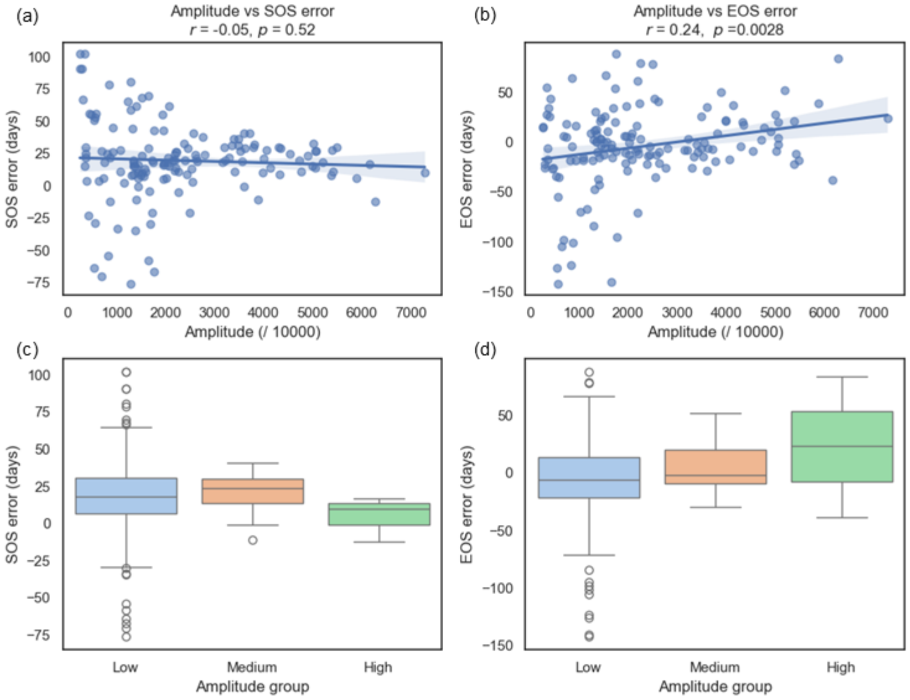

Figure S6. Seasonal amplitude vs. (a, b) SOS/EOS error scatter plots and linear regression for PhenoCam sites. And performance of (c, d) SOS/EOS errors across different seasonal amplitude groups.

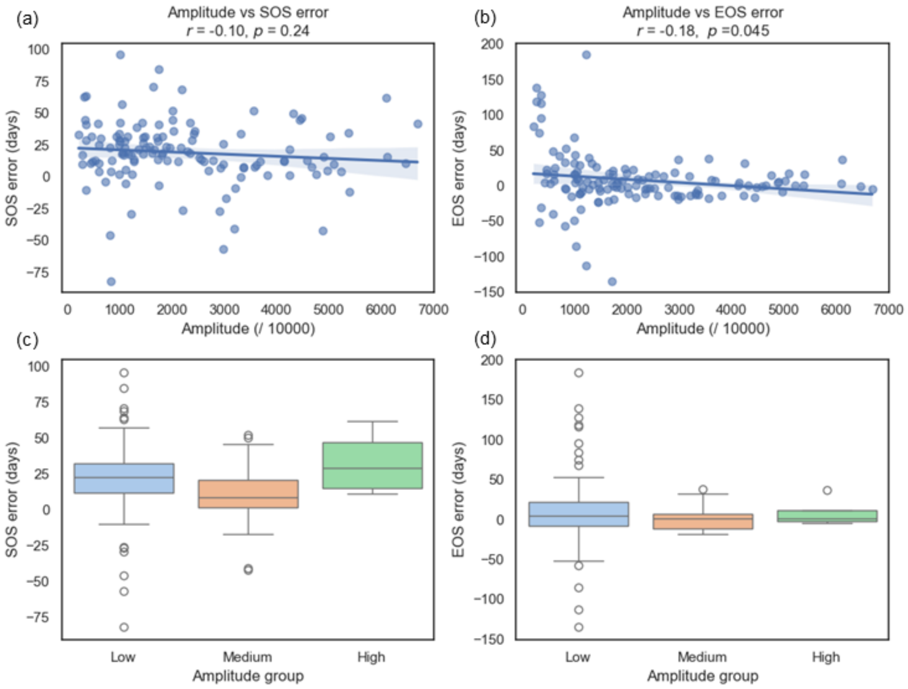

Figure S7. Seasonal amplitude vs. (a, b) SOS/EOS error scatter plots and linear regression for flux sites. And performance of (c, d) SOS/EOS errors across different seasonal amplitude groups.

Text S6. Evaluation of GDPD algorithm with VIIRS data

To further assess the robustness of the GDPD algorithm across different satellite sensors, we processed the 500-m daily VNP43IA4 Nadir BRDF-Adjusted Reflectance (NBAR) product to calculate EVI2 (consistent with the VNP22Q2 product), and applied the GDPD algorithm to generate phenological metrics. We then compared the VIIRS-based GDPD results with ground-based observations (PhenoCam data in Fig. S8 and flux tower data in Fig. S9), as well as with the MODIS-based GDPD results and the VNP22Q2 product. Since VIIRS data have only been available since 2012, all site data used in this analysis are after 2012.

The results demonstrate that phenological metrics derived from VIIRS using the GDPD algorithm exhibit high consistency with in-situ observations and provide broader coverage than the VNP22Q2 product. Specifically, at PhenoCam sites, the number of matched samples was 156 for VIIRS+GDPD (Fig. S8b), compared to 146 for VNP22Q2 (Fig. 4 in manuscript), with correlation coefficients of 0.79

for SOS (Fig. S8b) and 0.75 for EOS (Fig. S8f). At flux tower sites, VIIRS+GDPD matched 60 samples (Fig. S9b), while VNP22Q2 matched only 34 (Fig. 6 in manuscript), with correlation coefficients of 0.97 for SOS (Fig. S9b) and 0.88 for EOS (Fig. S9h).

Furthermore, we observed strong consistency between the MODIS- and VIIRS-based GDPD results during their overlap period (Fig. S8c, S8g, S9c, S9g), highlighting the robustness of the GDPD algorithm across sensors. For the PhenoCam sites (n = 292), the correlation between MODIS- and VIIRS-based GDPD results was $r = 0.85$ (RMSE = 30 days) for SOS and $r = 0.80$ (RMSE = 41 days) for EOS. For the flux tower sites (n = 148), the corresponding values were $r = 0.93$ (RMSE = 33 days) for SOS and $r = 0.81$ (RMSE = 65 days) for EOS.

These findings support the applicability of GDPD algorithm to future phenological monitoring as VIIRS increasingly replaces MODIS as the primary source of global optical remote sensing data.

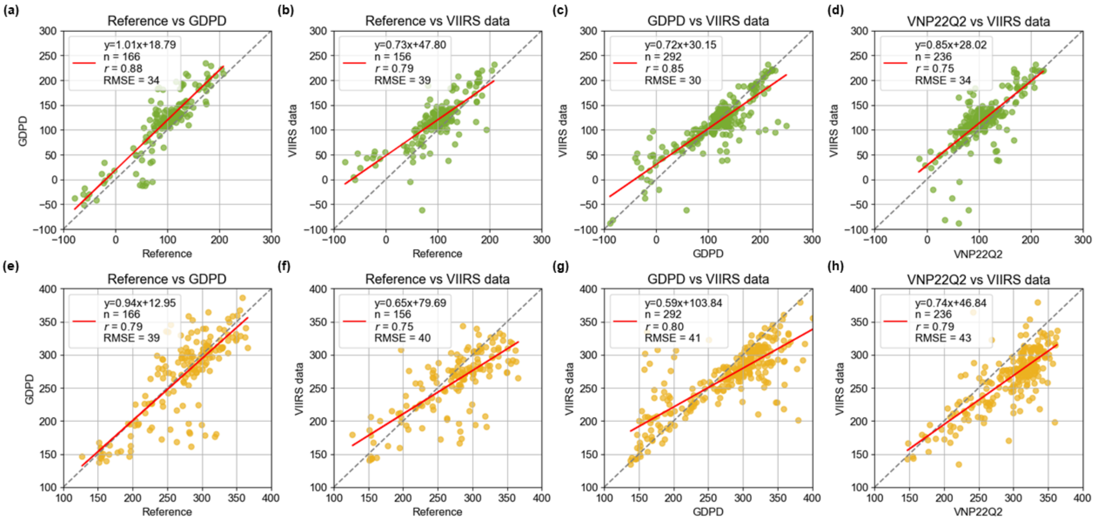

Figure S8. Comparison of phenological metrics between PhenoCam observations and (a, e) MODIS-based GDPD results, (b, f) VIIRS-based GDPD results; (c, g) comparison between MODIS-based and VIIRS-based GDPD results; and (d, h) comparison between VIIRS-based GDPD results and VNP22Q2. Green dots in panels (a–d) represent comparisons of start of season (SOS), and yellow dots in panels (e–h) represent comparisons of end of season (EOS). n indicates the number of matched samples; *r* is the Pearson correlation coefficient; RMSE is the root mean square error.

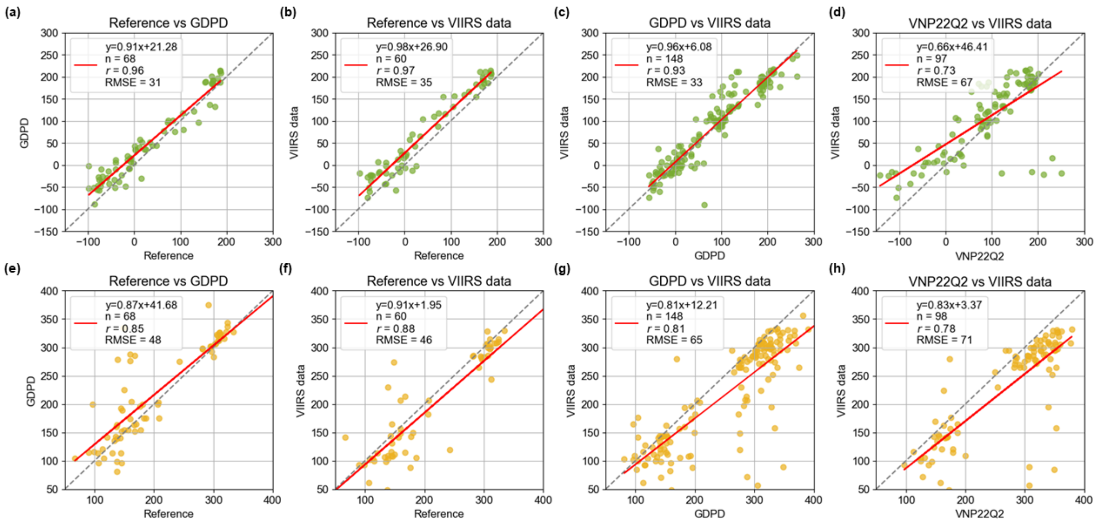

Figure S9. Comparison of phenological metrics between flux tower observations and (a, e) MODIS-based GDPD results, (b, f) VIIRS-based GDPD results; (c, g) comparison between MODIS-based and VIIRS-based GDPD results; and (d, h) comparison between VIIRS-based GDPD results and VNP22Q2. Green dots in panels (a–d) represent comparisons of start of season (SOS), and yellow dots in panels (e–h) represent comparisons of end of season (EOS). n indicates the number of matched samples; *r* is the Pearson correlation coefficient; RMSE is the root mean square error.

Table S5. List of PhenoCam sites used in accuracy assessment.

ID	Site name	Latitude	Longitude	Vegetation type	Start date	End date	Site years
1	arsgreatbasinltar098	43.17	–116.71	SHR	2017/5/17	2018/9/11	1.3
2	ashbottoms	38.87	–96.90	DBF	2015/8/18	2017/1/14	1.4
3	bozeman	45.78	–110.78	GRA	2016/11/12	2018/12/31	2.2
4	burnssagebrush	43.47	–119.69	SHR	2012/10/13	2018/12/31	6.2
5	butte	45.95	–112.48	GRA	2009/1/11	2018/12/31	10.0
6	canadaOA	53.63	–106.20	DBF	2011/6/16	2018/7/22	16.2
7	cperagm	40.84	–104.77	GRA	2016/5/19	2018/12/31	2.6
8	cpertgm	40.83	–104.76	GRA	2016/5/4	2018/12/31	2.7
9	cperuvb	40.81	–104.76	GRA	2015/7/16	2018/12/31	3.5
10	donanafuenteduque	37.00	–6.43	WET	2017/11/13	2018/12/31	1.2
11	freemangrass	29.93	–98.01	GRA	2012/3/14	2014/2/28	2.0
12	freemanwood	29.94	–97.99	DBF	2012/6/30	2014/3/21	1.7
13	grandteton	43.92	–110.58	SHR	2015/7/28	2018/12/31	3.5
14	ibp	32.59	–106.85	SHR	2014/2/16	2018/12/31	4.9
15	imcrktussock	68.61	–149.30	TUN	2012/7/9	2018/10/23	6.3
16	jasperridge	37.40	–122.22	GRA	2012/3/8	2018/12/31	6.8
17	jerbajada	32.58	–106.63	SHR	2014/4/20	2018/12/31	4.7
18	jernort	32.62	–106.79	SHR	2014/3/3	2018/12/31	4.9
19	jernwern	32.63	–106.74	SHR	2017/11/7	2018/12/31	1.2
20	jersand	32.52	–106.80	SHR	2014/2/28	2018/12/31	4.9

21	juncabalejo	36.94	–6.38	WET	2016/11/9	2018/12/31	2.2
22	kendall	31.74	–109.94	GRA	2012/7/6	2018/12/31	6.5
23	konza	39.08	–96.56	GRA	2012/3/17	2018/12/13	6.7
24	lethbridge	49.71	–112.94	GRA	2011/12/7	2018/12/31	7.1
25	luckyhills	31.74	–110.05	SHR	2013/4/29	2018/12/31	13.8
26	marena	36.06	–97.21	GRA	2012/6/12	2018/6/19	6.0
27	montebondonegrass	46.01	11.05	GRA	2015/4/29	2018/12/28	3.7
28	nationalelkrefuge	43.49	–110.74	GRA	2015/8/12	2018/12/31	3.4
29	NEON.D06.KING.DP1.20002	39.11	–96.60	WET	2017/9/2	2018/12/31	1.4
30	NEON.D06.KONZ.DP1.00033	39.10	–96.56	GRA	2017/2/25	2018/12/31	1.9
31	NEON.D09.NOGP.DP1.00033	46.77	–100.92	GRA	2017/10/29	2018/12/31	1.2
32	NEON.D09.PRLA.DP1.20002	47.16	–99.11	GRA	2017/10/29	2018/12/31	1.2
33	NEON.D10.ARIK.DP1.20002	39.76	–102.45	GRA	2016/12/18	2018/12/31	2.1
34	NEON.D10.CPER.DP1.00033	40.82	–104.75	GRA	2016/6/30	2018/12/31	2.5
35	NEON.D11.CLBJ.DP1.00033	33.40	–97.57	DBF	2017/2/13	2018/12/31	4.6
36	NEON.D11.OAES.DP1.00033	35.41	–99.06	GRA	2017/2/28	2018/12/31	1.9
37	NEON.D14.JORN.DP1.00033	32.59	–106.84	GRA	2017/2/25	2018/12/31	1.9
38	NEON.D15.ONAQ.DP1.00033	40.18	–112.45	SHR	2016/12/18	2018/12/31	2.1
39	quickbird	41.93	–109.30	SHR	2014/5/26	2015/12/16	1.6
40	sevilletagrass	34.36	–106.70	GRA	2014/11/7	2018/12/31	4.2
41	vaira	-120.95	38.41	GRA	2011/10/17	2018/12/31	7.2

Table S6. List of flux tower sites used in accuracy assessment. Data for the first 12 sites are from OzFlux, later sites are from FLUXNET 2015.

ID	Site ID	Site name	Latitude	Longitude	Vegetation type	Start year	End year	Years	Years_QC > 0.75
1	AU-ASM	Alice Springs	–22.28	133.25	SAV	2010	2020	11	/
2	AU-Cum	Cumberland Plain	–33.62	150.72	WSA	2012	2020	9	/
3	AU-DaP	Daly River Pasture	–14.06	131.32	GRA	2007	2013	7	/
4	AU-DaS	Daly River Uncleared	–14.16	131.39	SAV	2008	2014	7	/
5	AU-Dry	Dry River	–15.26	132.37	SAV	2008	2014	7	/
6	AU-GWW	Great Western Woodlands	–30.19	120.65	SAV	2013	2020	8	/
7	AU-Lon	Longreach Mitchell Grass Rangelands	–23.52	144.31	GRA	2018	2020	3	/
8	AU-RDF	Red Dirt Melon Farm	–14.56	132.48	WSA	2011	2013	3	/
9	AU-Rig	Riggs Creek	–36.65	145.58	GRA	2011	2014	4	/
10	AU-Stp	Sturt Plains	–17.15	133.35	GRA	2008	2014	7	/
11	AU-TTE	Ti Tree East	–22.29	133.64	GRA	2012	2019	8	/
12	AU-Ync	Yanco	–34.99	146.29	GRA	2012	2020	9	/
13	CA-Oas	Saskatchewan - Western Boreal, Mature Aspen	53.63	–106.20	DBF	2000	2010	11	11
14	CN-HaM	Haibei Alpine Tibet site	37.37	101.18	GRA	2002	2004	3	3
15	ES-LJu	Llano de los Juanes	36.93	–2.75	OSH	2004	2013	10	8
16	IT-MBo	Monte Bondone	46.01	11.05	GRA	2003	2013	11	11
17	IT-Ro1	Roccarespampani 1	42.41	11.93	DBF	2000	2008	9	7
18	IT-Ro2	Roccarespampani 2	42.39	11.92	DBF	2002	2012	10	8
19	US-SRC	Santa Rita Creosote	31.91	–110.84	OSH	2008	2014	7	4

20	US-SRG	Santa Rita Grassland	31.79	–110.83	GRA	2008	2014	7	7
21	US-SRM	Santa Rita Mesquite	31.82	–110.87	WSA	2004	2014	11	11
22	US-Var	Vaira Ranch- Ione	38.41	–120.95	GRA	2000	2014	15	14
23	US-Whs	Walnut Gulch Lucky Hills Shrub	31.74	–110.05	OSH	2007	2014	8	7
24	US-Wkg	Walnut Gulch Kendall Grasslands	31.74	–109.94	GRA	2004	2014	11	10

References

1. Gray, J., Sulla-Menashe, D. & Friedl, M. A. User Guide to Collection 6.1 MODIS Land Cover Dynamics (MCD12Q2) Product. (2022).
2. Zhang, X. *et al.* Evaluation of land surface phenology from VIIRS data using time series of PhenoCam imagery. *Agric. For. Meteorol.* **256–257**, 137–149 (2018).
3. Wu, W., Sun, Y., Xiao, K. & Xin, Q. Development of a global annual land surface phenology dataset for 1982–2018 from the AVHRR data by implementing multiple phenology retrieving methods. *Int. J. Appl. Earth Obs. Geoinformation* **103**, 102487 (2021).
4. Didan, K. & Barreto, A. NASA MEaSUREs Vegetation Index and Phenology (VIP) Phenology EVI2 Yearly Global 0.05Deg CMG [Data set]. https://doi.org/10.5067/MEaSUREs/VIP/VIPPHEN_EVI2.004 (2024).
5. Tian, F. *et al.* Calibrating vegetation phenology from Sentinel-2 using eddy covariance, PhenoCam, and PEP725 networks across Europe. *Remote Sens. Environ.* **260**, 112456 (2021).
